# Reduced NLRP3, AIM2 and NLRC4 inflammasome activity in cord blood of extremely preterm infants

**DOI:** 10.3389/fped.2026.1909501

**Published:** 2026-09-08

**Authors:** Hannah Siedel, Kristin Hieronymus, Jan M. Niehues, Lev Grinstein, Susanne Russ, Avril A. B. Robertson, Matthew A. Cooper, Jennifer L. Winkler, Angela Rösen-Wolff, Stefan Winkler

**Affiliations:** 1 Department of Pediatrics, University Hospital Carl Gustav Carus, Technische Universität Dresden, Dresden, Germany; 2 Department of Pediatrics, University Medical Center Hamburg-Eppendorf, Hamburg, Germany; 3 School of Chemistry and Molecular Biosciences, University of Queensland, Brisbane, QLD, Australia; 4 Institute for Molecular Bioscience, University of Queensland, Brisbane, QLD, Australia; 5 Sitala Bio Ltd., Cambridge, United Kingdom; 6 Department of Gynecology and Obstetrics, University Hospital Carl Gustav Carus, Technische Universität Dresden, Dresden, Germany

**Keywords:** caspase-1, cord blood, inflammasome, interleukin-1b, neonates, pre-term, whole blood assay

## Abstract

**Background:**

Pro-inﬂammatory caspase-1 is a key player in innate immunity. Physiological activation of the inflammasome/caspase-1 pathway supports host defense against pathogens, whereas dysregulated activation contributes to the pathogenesis of neonatal diseases such as bronchopulmonary dysplasia. Although preterm infants exhibit reduced IL-1β secretion from monocytes compared with term infants, a comprehensive functional characterization of distinct inflammasome pathways is lacking.

**Objective:**

To specifically analyze the activity of NLRP3, AIM2 and NLRC4 inflammasomes in cord blood of pre- and full-term infants and in peripheral blood of healthy adults.

**Methods:**

A validated whole-blood assay was used to specifically activate the different inflammasomes. IL-1β was analyzed as a surrogate marker of inflammasome/caspase-1 activation. RT-qPCR was used to measure expression of inflammasome related genes in leukocytes.

**Results:**

Activity of the NLRP3 and AIM2 inflammasomes was reduced in cord blood of preterm infants, particularly those born at <28 weeks of gestation, compared with term infants and adults. NLRC4 inflammasome activity was significantly reduced only in preterm infants with gestational age <32 weeks. No significant differences were observed between full-term infants and adult controls. Gene expression analysis revealed an age-dependent upregulation of NLRP3, AIM2, NLRC4, ASC, CASP1 and IL1B, with expression levels increasing from preterm to term gestation and even further towards adulthood.

**Conclusion:**

Activity of the inflammasome/caspase-1 pathway correlates with gestational age and is significantly reduced in preterm infants compared with full-term infants or adults. This finding contrasts with the high incidence of preterm-specific disorders where dysregulated caspase-1 activation is implicated in disease pathogenesis. Future research should investigate the dynamic evolution of inflammasome activity during the first days of life to elucidate its role in the onset of neonatal diseases.

## 1 Introduction

Pro-inflammatory caspase-1 signaling pathways are crucial for innate immune response, thereby contributing to pathogen clearance. Pathogens, stress and damage signals induce activation of caspase-1, typically mediated by proximity-induced autoproteolysis in multimeric protein complexes called inflammasomes. Active caspase-1 processes pro-Interleukin (IL)-1β and pro-IL-18 into their active forms and mediates pyroptosis, a programmed, proinflammatory cell death (1). Dysregulated activation of caspase-1 is involved in the pathogenesis of numerous diseases, from rare autoinflammatory syndromes to common diseases such as rheumatoid arthritis and gout (2, 3). In recent years, the caspase-1 signaling pathway has emerged as a key area of research in neonatal diseases, such as bronchopulmonary dysplasia (BPD), hypoxic ischemic encephalopathy (HIE) and neonatal sepsis (4).

BPD is a chronic lung disease typically affecting preterm infants (5). With over 13 million preterm births occurring each year worldwide, this population represents a major clinical and economic burden. However, the challenges of conducting research and obtaining samples from these vulnerable patients have limited our understanding of neonatal immune mechanisms and their role in disease pathogenesis. Hence, substantial research on the neonatal organism has been conducted using animal models. These studies have established the NLRP3 inflammasome as a key driver in BPD development (6, 7).

A limited number of studies have investigated the inflammasome/caspase-1 pathway in pre- and full-term infants. Sharma et al. reported impaired NLRP3 inflammasome activity in cord blood derived monocytes from preterm infants compared to term infants and adults (8). Similarly, Glaser et al. found reduced IL-1β release in response to Toll-like receptor (TLR) stimulation in monocytes from preterm infants, highlighting a functional deficit in innate immune activation at birth (9).

However, these studies exhibit some limitations. First, investigations were typically performed on purified monocytes or PBMCs, thereby excluding soluble plasma factors and cell-cell interactions that may modulate innate immune responses in whole blood (10–12). Whole-blood assays are intended to preserve these components and can therefore provide a more physiologically representative *ex vivo* model than assays based exclusively on purified cell populations. Second, caspase-1 is expressed not only in monocytes, macrophages and dendritic cells, but also in polymorphonuclear neutrophils (PMNs)—representing the major leukocyte subset in peripheral blood—which are typically absent from PBMC or monocyte preparations (13). Consequently, analyses performed in purified PBMCs or monocytes may not fully capture inflammasome–caspase-1 activity in the intact blood compartment. To our knowledge, no study has systematically evaluated NLRP3, AIM2, and NLRC4 inflammasome activity in whole blood from preterm and term infants.

In this study, we used a validated whole blood assay to analyze the activity of the NLRP3, AIM2 and NLRC4 inflammasomes in cord blood of pre- and full-term infants compared to adult peripheral blood (14). Caspase-1 activity and inflammasome related gene expression were analyzed following specific stimulation of each inflammasome pathway.

## 2 Materials and methods

### 2.1 Patients

Inclusion criteria for infants were inborn delivery and gestational age ≤ 36 + 6 weeks (group 1, preterm infants) or 37 + 0–42 + 0 weeks (group 2, full-term infants) (15, 16). Exclusion criteria for groups 1 and 2 were prolonged premature rupture of membranes > 24 h, signs of chorioamnionitis and neonatal asphyxia (umbilical artery pH < 7.0, 5-minute Apgar score ≤ 5). Inclusion criteria for the adult participants (group 3) were age ≥ 18 and < 50 years. Exclusion criteria for group 3 were any acute or chronic diseases and medical treatment. The study was conducted in compliance with the Declaration of Helsinki and approved by the responsible ethics committee (Ethics committee of the TU Dresden, EK97032014) before start. Blood samples for whole blood assays and complete blood counts were collected between June 2020 and August 2025. Blood samples for gene expression analysis were collected between June 2020 and May 2022. Cord blood samples were collected from the umbilical vein immediately after clamping of the umbilical cord. Peripheral blood samples were obtained from adults by venipuncture.

### 2.2 Whole blood assay

Hirudin coated tubes (Sarstedt 04.1944.001) were used to collect blood from patients. All whole-blood assays were initiated within 6 h of blood collection. Undiluted whole blood was aliquoted into 96-well plates at 140 µL per well. Priming for NLRP3- and AIM2-assays was performed using 10 ng/mL ultra-pure LPS (Invivogen, tlrl-3pelps) for 5.5 h. Plates were incubated on a shaker (450 rpm) in a humidified incubator with 37 °C, 5% CO2. For NLRP3 activation, additional incubation with 1 mM ATP for 30 min was performed. The activation of the AIM2 inflammasome was performed by transfection of double stranded DNA [poly(dA:dT), Invivogen, tlrl-patn]. Therefore, 0.8 μg Lipofectamine 2000 (ThermoFisher Scientific) and 0.4 μg poly(dA:dT) (Invivogen, tlrl-patn) were diluted each in a total volume of 10 μl in OptiMEM medium. Both volumes were mixed in a ratio of 1:1 and incubated for 5 min at room temperature. The total volume of 20 μl was added per well [final concentration 4 μg/mL Lipofectamine 2000, 2 μg/mL poly(dA:dT)] for 6 h. The NLRC4 inflammasome was stimulated with Salmonella typhimurium (strain SL1344) from an over-night culture in LB broth containing 300 mM NaCl and 200 μg/mL Streptomycin. Salmonella were washed with PBS twice. An estimated multiplicity of infection (MOI) of 10/leukocyte was used. Therefore, a leukocyte count of 10.0 × 10^9^/l was assumed for all patients. After 4 h of stimulation, 20 μg/mL Gentamicin was added to the cells followed by a second incubation step of 2 h. For NLRC4 activation, cells were not agitated during stimulations. A stock solution of MCC950 (10 mM)—synthesized by A.A.B.R. and M.A.C. as described earlier (17)—was dissolved in PBS and used at a final concentration of 10 µM. The inhibitor was added to whole blood samples together with the priming stimuli. The total volume for all inflammasome assays was 200 μl/well. At the end of all stimulations 100 μl of PBS was added to each well, the plates were centrifuged (1,200 rpm, 5 min, room temperature) and the supernatant from each well was frozen at−80 °C. The supernatants were analyzed within two weeks of collection. IL-1β concentrations in the supernatants were quantified using a bead-based immunoassay [BD™ Cytometric Bead Array (CBA) Human Soluble Protein Master Buffer Kit, catalog no. 558265, and BD™ CBA Human IL-1β Flex Set, catalog no. 558279; Becton, Dickinson and Company, Franklin Lakes, NJ, USA], according to the manufacturer's instructions. A standard curve was generated for each assay, and all samples were analyzed in duplicate. Fluorescence was measured using an LSR II flow cytometer, and data were analyzed with FCAP Array™ software (both from Becton, Dickinson and Company). Unless otherwise stated, data are presented as the mean ± SEM.

### 2.3 Blood count analysis

EDTA coated tubes (Sarstedt 20.1341) were used to collect blood from patients. Complete blood counts and leukocyte differentials were determined within 2 h of blood collection using an automated hematology analyzer (Sysmex XN 1000).

### 2.4 RNA isolation

PAXgene Blood RNA tubes (PreAnalytix, 762165) were used to collect blood from patients. Blood was stored at −20 °C until RNA isolation. RNA purification was performed within two years of sample collection. Total RNA was purified using the PAXgene Blood RNA kit (PreAnalytix, 762174). The manufacturer's protocol for manual isolation of total RNA from human whole blood collected in PAXgene Blood RNA tubes was strictly followed. In brief, total RNA was isolated from 2.5 mL of stabilized blood collected in PAXgene Blood RNA tubes, including on-column DNAse I digestion. Elution was performed using 2 × 40 µL of elution buffer. RNA samples were aliquoted and immediately stored at −80 °C. RNA concentration was measured using the QuantiFluor RNA System (Promega, E3310) with a Quantus Fluorometer (Promega, E6150), and RNA integrity was assessed by electrophoresis on a 1.2% denaturing agarose gel stained with ethidium bromide.

### 2.5 Gene expression analysis

cDNA synthesis was performed using M-MLV reverse transcriptase (Promega, M1701) within 6 months of RNA isolation. 1 μg total RNA was mixed with 0.5 μL random hexamer (Promega, C1181; 10 µM) and 0.5 μL oligo dT primer (Promega, C1101; 10 µM) and filled up with H_2_O to 25 μL reaction volume. After incubation at 70 °C for 10 min and chilling at 4 °C for 5 min, the reverse transcriptase reaction buffer (Promega, M531A), dNTPs, RNAsin Plus (Promega, N2611) and M-MLV reverse transcriptase was added according to the manufacturers’ instructions. Complete reaction volume was 35*μ*l. Transcription was performed at 42 °C for 60 min and reverse transcriptase was inactivated at 95 °C for 5 min. The cDNA obtained was aliquoted and stored at −20 °C until use.

For RT-qPCR, GoTaq qPCR Master Mix (Promega, A6002) was used according to the manufacturer's instructions with primer concentrations of 0.25 µM. PCR amplification was performed in a 20 µL reaction mixture containing 1 µL of cDNA, 10 µL of GoTaq® qPCR Master Mix, 0.5 µL each of the forward and reverse primers, 0.2 µL of CXR reference dye, and 7.8 µL of nuclease-free water.

RT-qPCR was run on the Applied Biosystems 7300 Real-Time PCR System in triplicates. PCR conditions included an initial linearization step at 95 °C for 10 min, followed by 40 cycles of denaturation at 95 °C for 15 s and annealing/extension at 60 °C for 1 min. Afterwards, the melting curve was analyzed to determine specificity of reaction products.

Primers were designed using Primer3 software via PrimerBlast (NCBI) and selected to produce amplicons spanning two exons. The primers were synthesized by Eurofins Genomics, Germany. Specificity was validated using cDNA from whole blood in endpoint PCR assays. PCR products were separated on a 2.5% agarose gel to validate expected size. The following primers were used: *CASP*1 5′-TTT CCG CAA GGT TCG ATT TTC A-3′ and 5′-GGC ATC TGC GCT CTA CCA TC-3′; *NLRP3* 5′-TAG CCA CGC TAA TGA TCG ACT-3′ and 5′-TTG ATC GCA GCG AAG ATC CAC-3′; *AIM2* 5′-TGG CAA AAC GTC TTC AGG AGG-3′ and 5′-AGCTTGACTTAGTGGCTTTGG-3′; *NLRC4* 5′-GAA CTG ATC GAC AGG ATG AAC G-3′ and 5′-ACC CAA GCT TGA CGA GTT GT-3′; *ASC* 5′-TGG ATG CTC TGT ACG GGA AG-3′ and 5′-CCA GGC TGG TGT GAA ACT GAA-3′; *IL1B* 5′-CAC GAT GCA CCC TGT ACG ATC A-3′ and 5′-GTT GCT CCA TAT CCT GTC CCT-3′; *ACTB* 5’-AGA GCT ACG AGC TGC CTG AC-3’ and 5’-AGC ACT GTG TTG GCG TAC AG-3’; *GAPDH* 5’-CCA TGA GAA GTA TGA CAA CAG CC-3’ and 5’-GGG TGC TAA GCA GTT GGT G-3’; *RPLP0* 5’-TGG CAG CAT CTA CAA CCC TG-3’ and 5’-ATC TGC AGA CAG ACA CTG GC-3’. Genes of interest were normalized to the geometric mean of the reference genes *ACTB* and *GAPDH* or to *RPLP0* as described earlier (18).

### 2.6 Statistical analysis

Inflammasome activity for NLRP3, AIM2 and NLRC4 inflammasomes was analyzed in preterm infants (group 1), full-term infants (group 2) and adult controls (group 3), including detailed subgrouping by gestational age and birth weight. Subgroups were defined using standard definitions for birth weight [extremely low birth weight (ELBW) < 1,000 g, very low birth weight (VLBW) 1,000 - < 1,500 g, low birth weight (LBW) 1,500–2,500 g, normal birth weight ≥2,500 g] or gestational age [extremely low gestational age (ELGA) < 28 + 0 weeks, very low gestational age (VLGA) 28 + 0–31 + 6 weeks, late preterm 32 + 0–36 + 6 weeks, and full-term ≥ 37 + 0] (15, 16). Differences in sample sizes between groups resulted in varying standard deviations within the smaller groups. Hence, the data of NLRP3, NLRC4 and AIM2 inflammasome activation were analyzed using Brown-Forsythe and Welch ANOVA test. Maternal and newborn group characteristics were calculated using Fisher's exact or Welch ANOVA test. Normalized gene expression was calculated from ct values using the 2^−*ΔΔ*ct^ method. Relative gene expression was normalized to the mean of the preterm infants (group 1) unless stated otherwise. Statistical analysis of gene expression was performed using one-way ANOVA comparing preterm infants, term infants and adults. Correlation analysis was performed using Spearman's rank correlation. *P*-values are defined as following: * = *p* < 0.05, ** = *p* < 0.01, *** = *p* < 0.001, **** = *p* < 0.0001. Outliers were identified and removed using ROUT method. Statistical analysis of patient characteristics was done using Fisher's exact test for multiple variables. Patient characteristics across subgroups were analyzed using Brown-Forsythe and Welch ANOVA tests. All statistical tests were performed using GraphPad Prism V11.0.2 except multivariable analysis.

For each inflammasome pathway, a separate multivariable linear regression analysis was performed using IBM SPSS Statistics version 31 to assess whether the association between gestational age and inflammasome activity was confounded by antenatal corticosteroid exposure, leukocyte count, or perinatal acidosis. Because IL-1β concentrations were strongly right-skewed (skewness 1.29–2.53; Kolmogorov–Smirnov and Shapiro–Wilk *p* < 0.001), values were natural-log-transformed to stabilize the variance and linearize the relationship with gestational age (Supplementary Table 12). Pearson correlation coefficients were calculated among gestational age, birth weight, white blood cell count (WBC), monocyte and neutrophil counts, antenatal corticosteroid category and umbilical artery pH to screen for collinearity. Gestational age and birth weight were near-collinear in all pathway datasets (r = 0.918–0.943, *p* < 0.001), such that only gestational age was retained as independent predictor. WBC was likewise strongly correlated with both monocyte and neutrophil counts (r = 0.82–0.96 across datasets, SupplementaryTable 10). To avoid collinearity among the leukocyte parameters, only total WBC was entered as a representative leukocyte predictor, while monocyte and neutrophil counts were analyzed in separate models. Antenatal corticosteroid exposure, although inversely correlated with gestational age (r = −0.72 to −0.81, *p* < 0.001), was deliberately retained as a categorical predictor (none/incomplete course/full course; dummy-coded with full course as reference), because corticosteroids and gestational maturity may exert distinct effects on innate immune function. The resulting collinearity was reported using variance inflation factors (VIF). Linear regression was used to model ln(IL-1β), entering gestational age, WBC and the corticosteroid dummies, and repeated with umbilical artery pH added as a further independent predictor. Diagnostics across resulting models showed that standardized residuals were approximately normally distributed (mean approx. 0, standard deviation approx. 0.95) with largely symmetric ranges, and the normal P–P plots of the standardized residuals aligned closely with the diagonal (Supplementary Figure 4).

## 3 Results

### 3.1 Patient characteristics

A total of 236 patients were included into the study and assigned to three groups based on their age: 87 preterm infants (group 1), 72 full-term infants (group 2), and 77 healthy adult controls (group 3). Due to practical constraints and limited blood volume, not all assays could be performed for every patient. The mean gestational age was 30 + 6 weeks for group 1 and 38 + 3 weeks for group 2 (Table 1). Exposure to antenatal corticosteroids existed only in group 1. The mode of delivery was caesarean section for all analyzed samples. According to local protocol, preterm infants < 34 weeks of gestation were delivered via cesarean section. The indications for cesarean delivery of full-term infants (group 2) were repeat cesarean section or previous abdominal surgery (52%), breech presentation (26%), cephalopelvic disproportion (6%), placenta previa (8%), pre-eclampsia (1%), pathological umbilical doppler or cardiotocography (4%) and uterine myomatosis (3%). The reasons for preterm birth were spontaneous preterm labor (22%), premature rupture of membranes (18%), pathological umbilical doppler or cardiotocography (30%), placenta previa with vaginal bleeding (1%), maternal psychological indication (1%), polyhydramnios of unknown origin (1%), cord or membrane prolapse (2%), monochorionic-monoamniotic pregnancy (6%), pre-eclampsia (4%) or hemolysis, elevated liver enzymes, low platelet count (HELLP) syndrome (15%) (Supplementary Table 1). None of the infants included in the study developed bacterial infection within 72 h after birth. The mean age of group 3 was 28.2 years (range 21–49) and the sex ratio was 68% female and 32% male. The adult controls did not show any clinical signs of infectious disease, did not suffer from acute or chronic diseases, and were not being treated with any long-term medication.

**Table 1:** Maternal and newborn characteristics by group.

Measure	Preterm infants (group 1, *n* = 87)	Full-term infants (group 2, *n* = 72)	*p*-value
Maternal age, mean (range), years	31.8 (19–47)	34.5 (25–42)	0.03
Gestational age, mean (range) in weeks + days	30 + 6 (23 + 2–36 + 6)	38 + 3 (37 + 0–41 + 3)	< 0.0001
Birth weight, mean (range), g ELBW (<1,000 g), *n* (%) VLBW (1,000–1,500 g), *n* (%) LBW (<2,500 g), *n* (%) ≥2,500 g, *n* (%)	1468 (350–3715) 23 (26) 28 (32) 30 (35) 6 (7)	3245 (1970–4570) 0 (0) 0 (0) 9 (13) 63 (87)	< 0.0001
Birth weight <10. Perc., *n* (%)	23 (26)	16 (22)	0.58
Infant sex			0.63
Female, *n* (%) Male, *n* (%)	41 (47) 46 (53)	31 (43) 41 (57)	
Receipt of antenatal corticosteroids			< 0.0001
None, *n* (%) Incomplete course, *n* (%) Full course, *n* (%)	22 (25) 9 (10) 56 (64)	72 (100) 0 (0) 0 (0)	
Umbilical artery pH, mean (range)	7.32 (7.13–7.47)	7.32 (7.21–7.39)	0.95
APGAR Score 10 min, mean, (range)	8.8 (5–10)	9.8 (8–10)	< 0.0001

### 3.2 Alternative and canonical NLRP3 inflammasome activity is reduced in preterm infants

**Figure 1:**
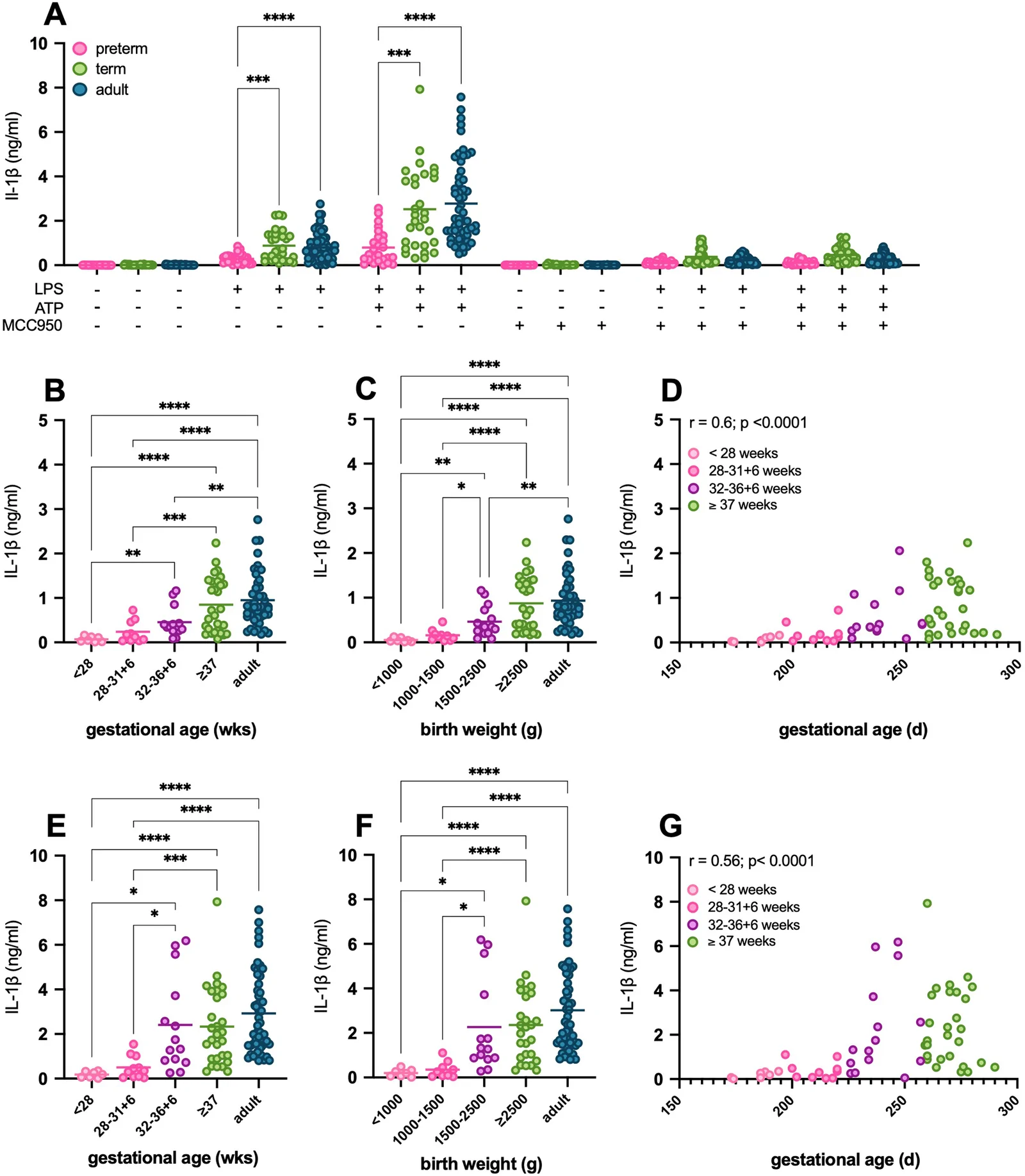
Activity of the NLRP3 inflammasome is reduced in preterm infants. Cord blood of pre- and full-term infants and peripheral blood of healthy adult controls were stimulated with LPS **(A–D)** or LPS and ATP **(A,E–G)**. IL-1β concentrations in the supernatant were analyzed as surrogate marker of inflammasome/caspase-1 activation. **(A)** Data were analyzed for groups 1 (*n* = 34), 2 (*n* = 32), and 3 (*n* = 52). For improved clarity, statistical analyses are presented only for LPS- or LPS/ATP-stimulated samples. **(B–D)** Stimulation of the alternative NLRP3 inflammasome with LPS. **(E–G)** Stimulation of the canonical NLRP3 inflammasome with LPS and ATP. Data were analyzed for all infants based on subgrouping for gestational age **(B,E)** (<28 + 0: *n* = 6, 28 + 0–31 + 6: *n* = 12, ≥32 + 0: *n* = 16) or birth weight **(C,F)** (<1,000g: *n* = 7, 1,000–1,500g: *n* = 11, 1,500–2,500g: *n* = 16, ≥2,500g: *n* = 32), as well as correlation to gestational age **(D,G)**. Statistical analysis was performed using Brown-Forsythe and Welch ANOVA test, correlation was analyzed using Spearman's rank correlation. Detailed information on sample sizes and group characteristics is provided in Supplementary Tables 2, 3.

Priming of the samples with LPS resulted in immediate IL-1β secretion across all groups. This effect is mediated by the alternative NLRP3 inflammasome, a constitutive active pathway specific to human monocytes (14, 19). Additional stimulation with ATP led to further increase of IL-1β secretion due to canonical NLRP3 activation (Figure 1A). For both pathways, IL-1β secretion was reduced in preterm infants compared to full-term infants and adults (Figure 1A). However, no difference in IL-1β secretion could be detected between full-term infants and adult controls. The NLRP3 specific inhibitor MCC950 strongly reduced NLRP3-mediated IL-1β secretion across all groups (Figure 1A). Detailed subgrouping of all infants based on existing definitions for gestational age (Figures 1B,E) or birth weight (Figures 1C,F) showed a strong reduction of IL-1β secretion towards lower gestational age or birth weight. Especially for infants born earlier than 28 weeks of gestation or with a birth weight <1,000 g, IL-1β secretion was extremely reduced. While IL-1β secretion following activation of the alternative NLRP3 pathway was reduced for all subgroups of preterm infants when compared to full-term infants, IL-1β secretion following stimulation of the canonical NLRP3 pathway was preserved for the group of late preterm infants (32 + 0–36 + 6 weeks of gestational age). The correlation between gestational age and IL-1β secretion could be confirmed for both pathways using Spearman's correlation analysis (Figures 1D,G).

### 3.3 AIM2 inflammasome activity is reduced in preterm infants

**Figure 2:**
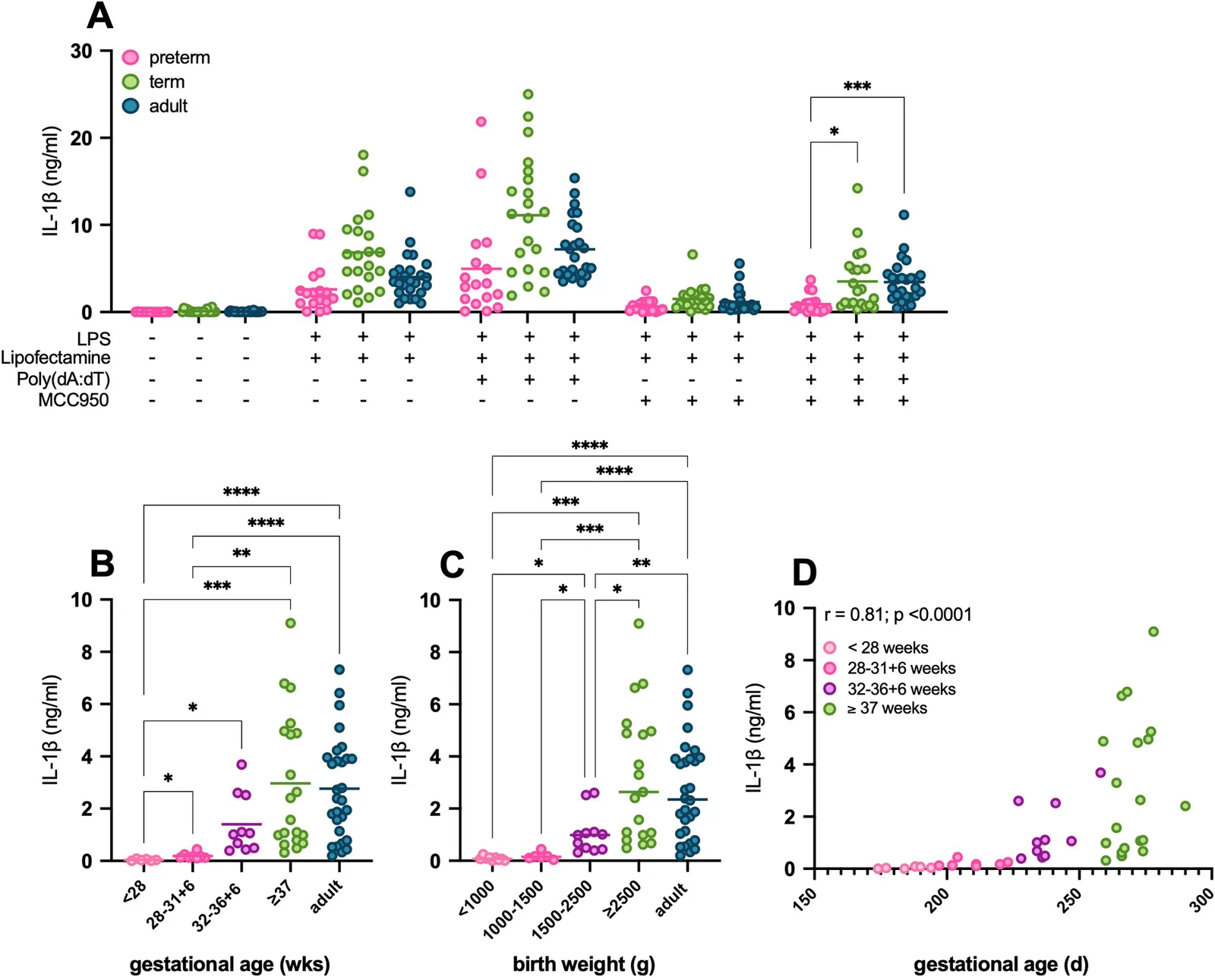
Activity of the AIM2 inflammasome is reduced in preterm infants. Cord blood of pre- and full-term infants and peripheral blood of healthy adults were stimulated with LPS and transfection of poly(dA:dT) in pre- or absence of the NLRP3 inhibitor MCC950. IL-1β concentrations in the supernatant were analyzed as surrogate marker of inflammasome/caspase-1 activation. **(A)** Data were analyzed for groups 1 (*n* = 28), 2 (*n* = 21), and 3 (*n* = 30). For improved clarity, statistical analyses are presented only for LPS-stimulated samples transfected with poly(dA:dT) in presence of MCC950. **(B–D)** The AIM2 inflammasome was stimulated with LPS and transfection of poly(dA:dT), while blocking NLRP3 activation using MCC950. Data were analyzed for all infants based on subgrouping for gestational age **(B)** (<28 + 0: *n* = 6, 28 + 0–31 + 6: *n* = 8, ≥32 + 0: *n* = 13) or birth weight **(C)** (<1,000g: *n* = 9, 1,000–1,500g: *n* = 6, 1,500–2,500g: *n* = 12, ≥2,500g: *n* = 23), as well as correlation to gestational age **(D)** Statistical analysis was performed using Brown-Forsythe and Welch ANOVA test, correlation was analyzed using Spearman's rank correlation. Detailed information on sample sizes and group characteristics is provided in Supplementary Tables 4, 5.

Activation of the AIM2 inflammasome was induced via LPS priming and additional transfection of synthetic double-stranded DNA (dsDNA) using Lipofectamine. This led to IL-1β secretion across all groups (Figure 2A). To solely analyze the AIM2 signal, we blocked NLRP3 activation using MCC950. Here, IL-1β secretion was reduced in preterm infants compared to full-term infants and adults (Figure 2A). However, no difference in IL-1β secretion could be detected between full-term infants and adult controls. In contrast, LPS primed samples, stimulated with Lipofectamine or Lipofectamine and dsDNA without MCC950 showed an enhanced secretion of IL-1β in the group of full-term infants compared to preterm infants or adult controls.

Detailed subgrouping of all infants based on WHO definitions for gestational age (Figure 2B) or birth weight (Figure 2C) showed a further reduction of IL-1β secretion towards lower gestational age or birth weight. Especially for infants born earlier than 28 weeks of gestation or with a birth weight <1,500 g, IL-1β secretion was extremely reduced. The positive correlation between gestational age and IL-1β secretion could be confirmed for the AIM2 pathway using Spearman's correlation analysis (Figure 2D).

### 3.4 Activity of the NLRC4 inflammasome is reduced in very low gestational age infants

**Figure 3:**
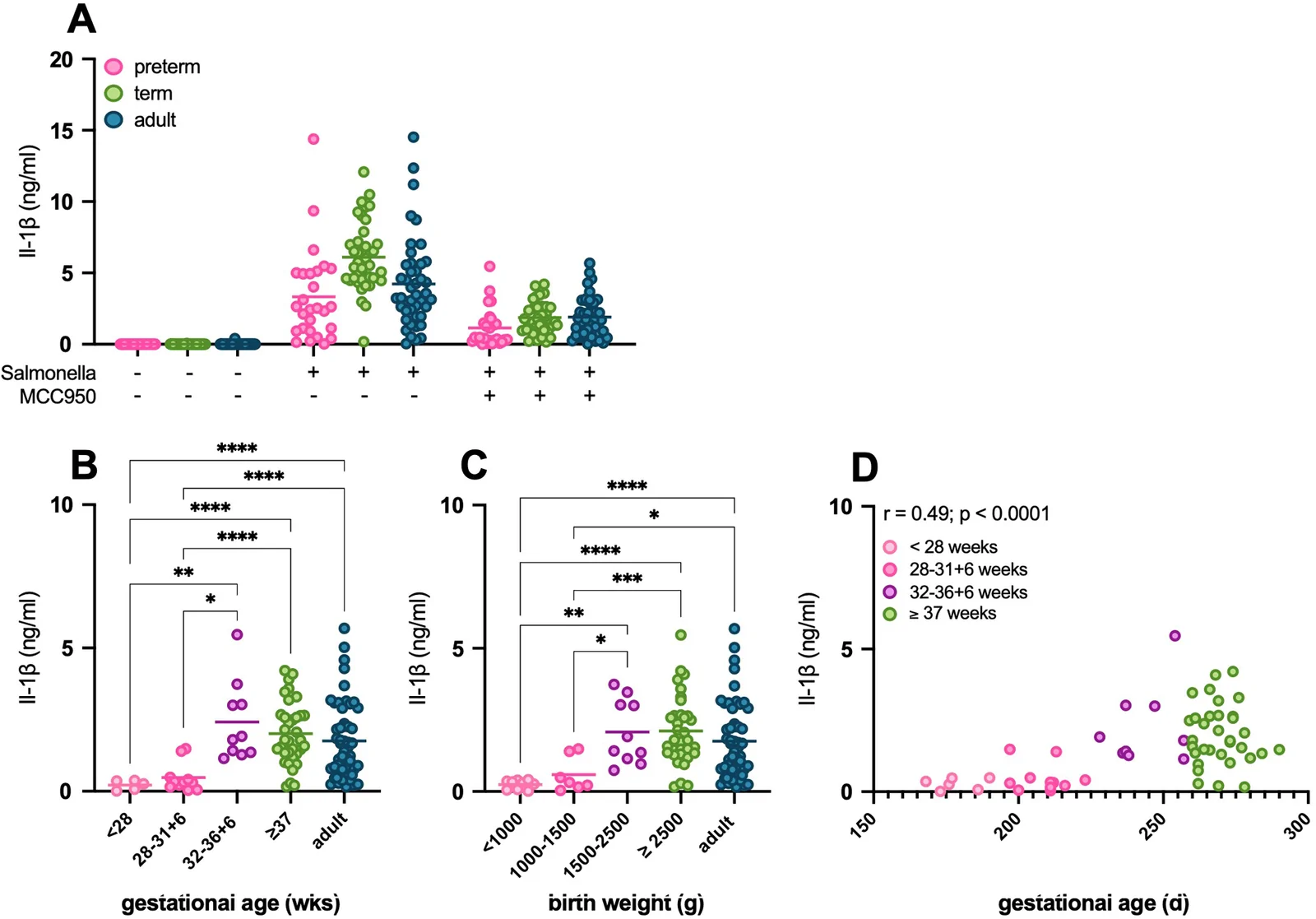
Activity of the NLRC4 inflammasome is reduced in very low gestational age infants. Cord blood of pre- and full-term infants and peripheral blood of healthy adults were stimulated with Salmonella typhimurium in pre- or absence of the NLRP3 inhibitor MCC950. IL-1β concentrations in the supernatant were analyzed as surrogate marker of inflammasome/caspase-1 activation. **(A)** Data were analyzed for groups 1 (*n* = 29), 2 (*n* = 39), and 3 (*n* = 57). **(B–D)** The NLRC4 inflammasome was stimulated with Salmonella typhimurium while blocking NLRP3 activation using MCC950. Data were analyzed for all infants based on subgrouping for gestational age **(B)** (<28 + 0: *n* = 5, 28 + 0–31 + 6: *n* = 13, ≥32 + 0: *n* = 11) or birth weight **(C)** (<1000g: *n* = 10, 1,000–1,500g: *n* = 8, 1,500–2,500g: *n* = 10, ≥2,500g: *n* = 40), as well as correlation to gestational age **(D)** Statistical analysis was performed using Brown-Forsythe and Welch ANOVA test, correlation was analyzed using Spearman's rank correlation. Detailed information on sample sizes and group characteristics is provided in Supplementary Tables 6, 7.

Stimulation of samples with *Salmonella typhimurium* led to IL-1β secretion across all groups (Figure 3A). A tendency of enhanced IL-1β secretion for the full-term infants compared to preterm infants or adult controls was found. As *S. typhimurium* mediated activation of Toll-like receptor 4 (TLR4) is known to induce IL-1β secretion via the alternative NLRP3 pathway, MCC950 was used to block NLRP3 and specifically analyze NLRC4 activation. In contrast to the NLRP3 or AIM2 inflammasome no statistically significant differences in IL-1β secretion were found between groups 1–3. However, detailed subgrouping of all infants based on WHO definitions for gestational age (Figure 3B) or birth weight (Figure 3C) revealed a reduction of IL-1β secretion for infants born earlier than 32 weeks of gestation or with a birth weight <1,500 g. Spearman's correlation analysis confirmed the correlation between gestational age and IL-1β secretion for the NLRC4 pathway (Figure 3D).

### 3.5 Gestational age is the most consistent predictor of inflammasome activity across the investigated pathways

**Figure 4:**
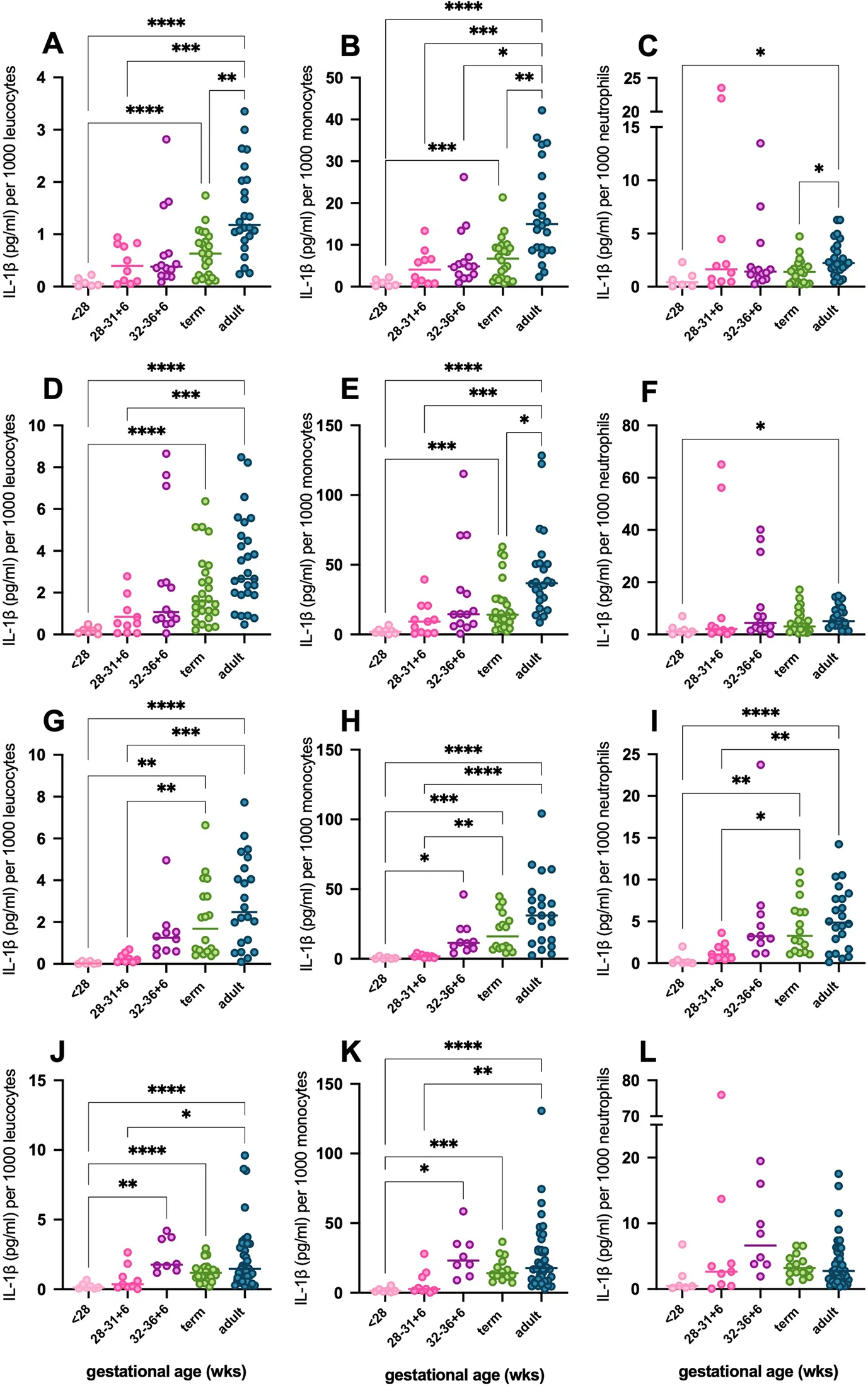
IL-1β concentrations normalized to cell counts. **(A–L)** Cord blood from preterm and term infants and peripheral blood from healthy adult controls were stimulated to activate the respective inflammasome pathways. IL-1β concentrations in the culture supernatants were measured as a surrogate marker of inflammasome/caspase-1 activation and normalized to the corresponding cell count for each sample. **(A–C)** Stimulation of the alternative NLRP3 inflammasome with LPS. IL-1β was normalized to the cell count of **(A)** total WBC, **(B)** monocytes, or **(C)** neutrophils. Data were analyzed for all infants based on subgrouping for gestational age [<28 + 0: *n* = 6, 28 + 0–31 + 6: *n* = 12, ≥32 + 0: *n* = 16, group 2 (*n* = 32), and 3 (*n* = 52)]. **(D–F)** Stimulation of the canonical NLRP3 inflammasome with LPS and ATP. IL-1β was normalized to the cell count of **(D)** total WBC, **(E)** monocytes, or **(F)** neutrophils. Data were analyzed for all infants based on subgrouping for gestational age [<28 + 0: *n* = 6, 28 + 0–31 + 6: *n* = 12, ≥32 + 0: *n* = 16, group 2 (*n* = 32), and 3 (*n* = 52)]. **(G–I)** Stimulation of the AIM2 inflammasome with LPS and transfection of poly(dA:dT) in presence of the NLRP3 inhibitor MCC950. IL-1β was normalized to cell count of **(G)** total WBC, **(H)** monocytes, or **(I)** neutrophils. Data were analyzed for all infants based on subgrouping for gestational age [<28 + 0: *n* = 6, 28 + 0–31 + 6: *n* = 8, ≥32 + 0: *n* = 13, group 2 (*n* = 21), and 3 (*n* = 30)]. **(J–L)** Stimulation of the NLRC4 inflammasome with LPS and Salmonella typhimurium in presence of the NLRP3 inhibitor MCC950. IL-1β was normalized to cell count of **(J)** total WBC, **(K)** monocytes, or **(L)** neutrophils. Data were analyzed for all infants based on subgrouping for gestational age [<28 + 0: *n* = 5, 28 + 0–31 + 6: *n* = 13, ≥32 + 0: *n* = 11, group 2 (*n* = 39), and 3 (*n* = 57)]. Statistical analysis was performed using Brown-Forsythe and Welch ANOVA test. Detailed information on sample sizes and group characteristics is provided in Supplementary Tables 2–7.

To assess whether total leukocyte count or differences in leukocyte composition influenced the results, total WBC, monocyte, and neutrophil counts were analyzed for each sample (Supplementary Figure 2). Because statistically significant differences were observed between the subgroups, IL-1β concentrations were subsequently normalized to the respective cell counts (Figure 4). Normalization to total WBC and monocyte counts preserved the age-dependent increase in IL-1β secretion and additionally revealed statistically significant differences between term infants and adult controls for both NLRP3 pathways. In contrast, normalization to neutrophil counts attenuated the age-dependent increase in IL-1β secretion, except for the AIM2 pathway.

Additional subgroup analyses were performed according to antenatal corticosteroid exposure status: no exposure, incomplete exposure, or a complete course of antenatal corticosteroids (Supplementary Figure 3). Among preterm infants exposed to a complete course of antenatal corticosteroids, a statistically significant age-dependent increase in IL-1β secretion was observed for the alternative NLRP3 and AIM2 pathways. However, the small sample size limited subgroup analyses. Nevertheless, the overall pattern of increasing IL-1β secretion with increasing age remained evident.

To assess confounding by antenatal corticosteroids, leukocyte count or perinatal acidosis, ln-transformed IL-1β was additionally modelled by multiple linear regression in the combined infant cohort (groups 1 and 2) for all four inflammasome pathways (Table 2). For both the alternative and canonical NLRP3 inflammasome, the overall models were statistically significant, with gestational age being the only significant independent predictor (Table 2). WBC, corticosteroid exposure category and umbilical artery pH were not significant. The strongest model fit was observed for the AIM2 inflammasome, in which gestational age was the predominant predictor. Here, the coefficient for no antenatal corticosteroid exposure reached the threshold for statistical significance, whereas WBC count, incomplete corticosteroid exposure, and umbilical artery pH were not significant. For the NLRC4 inflammasome the overall model was statistically significant, although none of the individual predictors reached statistical significance. Gestational age showed the strongest, but non-significant, association, whereas no significant effects were observed for WBC count, corticosteroid exposure category, or umbilical artery pH. Across all four inflammasomes, gestational age was the most consistent independent predictor of ln(IL-1β). In contrast, antenatal corticosteroid exposure showed no consistent independent association; the only nominally significant corticosteroid-related finding was observed for no antenatal corticosteroid exposure in the AIM2 model. Because corticosteroid exposure was strongly inversely correlated with gestational age (Supplementary Table 10), gestational age and the corticosteroid dummies showed moderately elevated variance inflation (Table 2). Gestational age and corticosteroid effects can therefore not be fully separated statistically. Substituting monocyte or neutrophil counts for total WBC count did not alter the conclusions regarding the effect of leukocyte count across the four inflammasome pathways (Supplementary Table 11).

**Table 2:** Multiple linear regression analysis predicting IL-1β. B, unstandardized coefficient; *β*, standardized coefficient; VIF, variance inflation factor. ANS, antenatal corticosteroids (reference category: full course). .

Pathway	n	R^2^	Predictor	B	*β*	p	VIF
Alternative NLRP3	49	.410	Gestational age	.032	.749	<0.001	2.98
F₅,₄₃ = 5.97, *p* < 0.001			WBC	.037	.169	.177	1.10
			ANS none	−.813	−.311	.124	2.86
			ANS incomplete	−.465	−.086	.496	1.13
			Umbilical artery pH	1.467	.059	.631	1.10
Canonical NLRP3	50	.418	Gestational age	.030	.593	.005	3.02
F₅,₄₄ = 6.31, *p* < 0.001			WBC	.017	.063	.604	1.11
			ANS none	−.256	−.082	.677	2.92
			ANS incomplete	−1.281	−.197	.113	1.12
			Umbilical artery pH	1.381	.047	.696	1.08
AIM2	38	.783	Gestational age	.065	1.136	<0.001	4.00
F₅,₃₂ = 23.06, *p* < 0.001			WBC	.022	.082	.341	1.06
			ANS none	−1.179	−.319	.050	3.63
			ANS incomplete	.361	.060	.523	1.28
			Umbilical artery pH	−4.315	−.159	.065	1.02
NLRC4	54	.438	Gestational age	.017	.462	.078	5.62
F₅,₄₈ = 7.50, *p* < 0.001			WBC	.036	.156	.180	1.12
			ANS none	.006	.002	.993	5.46
			ANS incomplete	−1.081	−.232	.063	1.28
			Umbilical artery pH	2.976	.149	.180	1.02

Overall, these findings do not indicate that differences in antenatal corticosteroid exposure, leukocyte count, or umbilical artery pH explained the reduced inflammasome activity observed in preterm infants.

### 3.6 Expression of inflammasome related genes is reduced in infants

**Figure 5:**
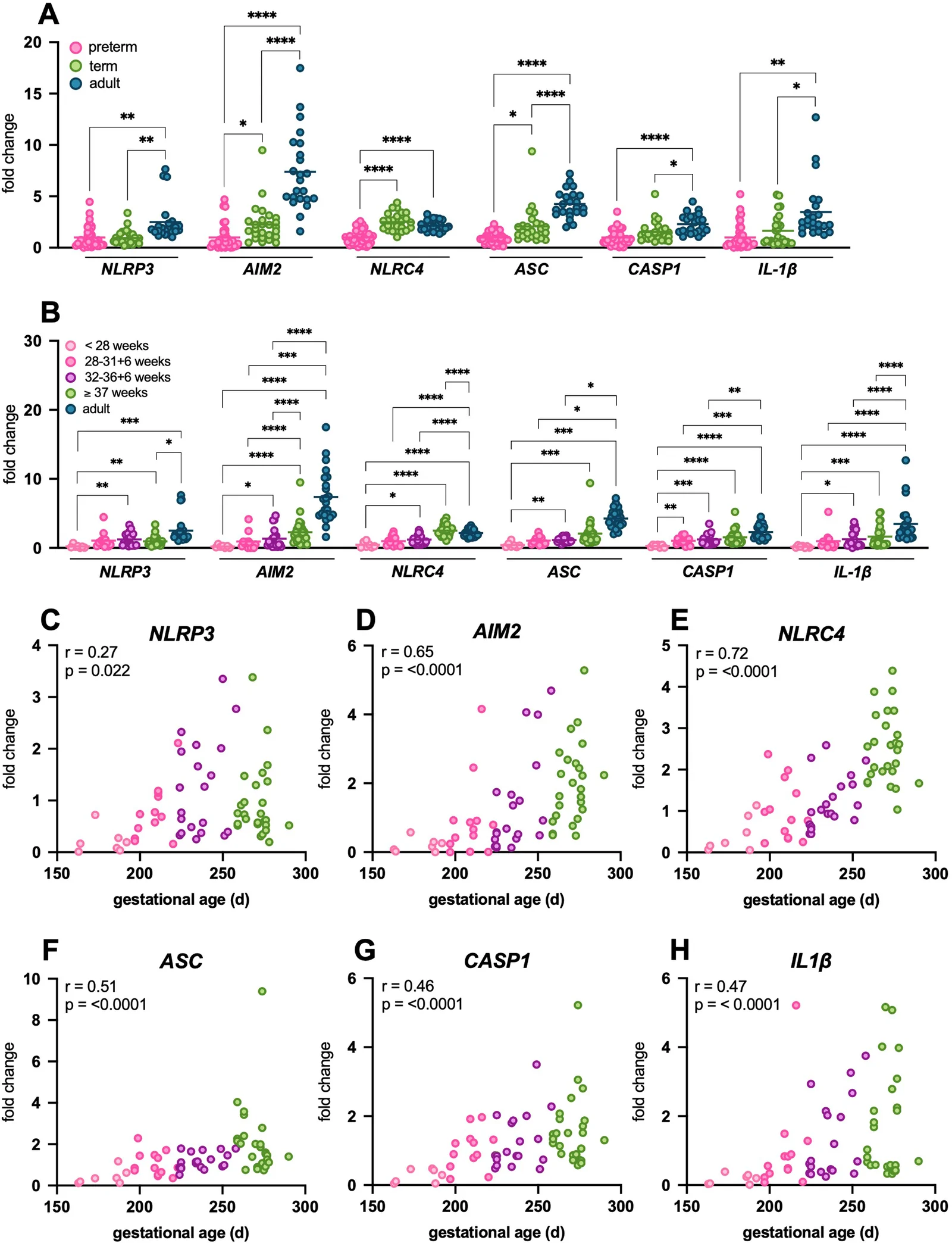
Expression of inflammasome related genes is reduced in infants. **(A–H)** Non-stimulated, stabilized cord blood samples from pre- and full-term infants and peripheral blood of healthy adults were analyzed for gene expression of NLRP3, AIM2, NLRC4, ASC, CASP1, and IL1*β* using qPCR. Expression of target genes was normalized to the geometric mean of the reference genes ACTB and GAPDH. **(A)** Data were analyzed for groups 1 (*n* = 40), 2 (*n* = 26), and 3 (*n* = 23). **(B)** Data were analyzed for all infants based on gestational age subgrouping (<28 + 0: *n* = 7, 28 + 0–31 + 6: *n* = 13, ≥32 + 0: *n* = 20) using Brown-Forsythe and Welch ANOVA test, and the correlation between gene expression and gestational age was assessed using Spearman's rank correlation. **(C–H)**. Detailed information on sample sizes and group characteristics is provided in Supplementary Tables 8, 9.

Owing to the markedly reduced activity of NLRP3, AIM2, and NLRC4 inflammasomes in preterm infants relative to full-term infants and adults, we aimed to elucidate the underlying mechanisms. Because pathway activity is frequently modulated at the transcriptional level, we analyzed the expression of six inflammasome-related genes *NLRP3*, *AIM2*, *NLRC4, ASC, CASP1* and *IL1β* in stabilized blood samples from groups 1–3. Expression of *AIM2, NLRC4,* and *ASC* was significantly higher in full-term infants compared to preterm infants (Figure 5A). *NLRP3*, *AIM2*, *ASC, CASP1* and *IL1β* showed an enhanced expression in adults when compared to full-term infants. Detailed subgrouping of preterm infants based on their gestational age showed an enhanced gene expression towards higher gestational age within group 1 (Figure 5B). Additionally, differential gene expression between at least one subgroup of preterm infants and the group of full-term infants could be proven for all genes analyzed. Spearman's correlation analysis revealed a positive correlation between gestational age and expression of all analyzed genes (Figures 5C–H). Gene expression data was normalized to an *ACTB*/*GAPDH* based normalization factor, as evaluated for whole blood assays earlier (18). An additional gene expression analysis normalized to *RPLP0* as a single reference gene confirmed these results (Supplementary Figure 1). Overall, the expression of all analyzed genes increased with age toward adulthood.

## 4 Discussion

Previous studies demonstrate an impaired production of IL-1β in the blood of preterm infants, but the specific inflammasome pathways have not been analyzed in detail (20–24). In the present study, we specifically analyzed the activity of the NLRP3, AIM2 and NLRC4 inflammasomes in cord blood of pre- and full-term infants and peripheral blood of healthy adults using a validated whole blood assay (14). We demonstrate a significant reduced inflammasome activity for ELGA/ELBW infants as well as a positive correlation between gestational age and activity of all three inflammasomes analyzed. The reduced activity of the canonical and alternative NLRP3 pathways for preterm and especially ELGA infants are consistent with previously published *in vitro* data (8, 9, 25). While most studies used purified monocytes for their functional analyses we can prove these findings in whole blood. This is important as plasma-factors or cellular interactions in whole blood are known to mediate immune cell function (10–12, 26). In addition, our findings also demonstrate reduced activation of the AIM2 and NLRC4 inflammasomes in preterm cord blood. Hence, the impairment of caspase-1 activation in early preterm infants extends across all three inflammasome pathways analyzed.

### 4.1 Patient characteristics and generalizability of results

The complex interplay of factors influencing fetal/neonatal gene expression, particularly in the context of preterm delivery and associated treatments, necessitates careful consideration when investigating the neonatal immune system. Key factors include sex, fetal growth restriction, antenatal corticosteroid exposure, the etiology of preterm delivery, and the mode of delivery (27–30). Sex distribution, frequency of fetal growth restriction, and mode of delivery did not differ between both groups of infants (groups 1 and 2) and are comparable to those reported in clinical trial cohorts (31, 32). The causes of preterm birth in the current study were comparable to those observed in larger cohorts of preterm infants, including spontaneous preterm labor (with or without premature rupture of membranes) and maternal/fetal indications for delivery (33, 34). The patient characteristics of infants in groups 1 and 2, despite this being a single-center study, were similar to those reported in larger clinical trials, indicating potential generalizability of the results.

### 4.2 Influence of antenatal corticosteroids on inflammasome pathways

Antenatal corticosteroid treatment is a cornerstone intervention for anticipated preterm delivery, significantly reducing the risk of respiratory distress syndrome and mortality in premature infants. Beyond their established benefits, antenatal corticosteroids may also induce adverse long-term health outcomes by modulating gene expression (28, 35). Unlike full-term infants, populations of preterm infants included in clinical studies typically have high coverage of antenatal steroid exposure (31, 32, 36). This intergroup difference, also observed in neonatal groups 1 and 2 of this study, may introduce experimental bias in the assessment of inflammasome/caspase-1 pathway activity. Although a study analyzing the impact of antenatal corticosteroids on gene expression in neonatal peripheral blood leukocytes found no direct influence on the differential expression of inflammasome-related genes, corticosteroids are known to exert a complex, context-dependent effect on inflammasome/caspase-1 pathways (28). The main reported effect of corticosteroids on the NLRP3 inflammasome is inhibition of NLRP3 activation, achieved through negative regulation of NLRP3 mRNA expression or interference with inflammasome priming (37, 38). In contrast, other studies report that glucocorticoids sensitize the NLRP3 inflammasome not only in human and murine macrophages but also in human monocyte-derived dendritic cells (39, 40). Sharma et al., however, reported no effect of antenatal corticosteroids on alternative NLRP3 inflammasome activation (22). For the AIM2 inflammasome, dexamethasone was reported to have no effect on this pathway (41), while data on the effect of corticosteroids on the NLRC4 inflammasome are currently lacking. Regarding our data, we cannot exclude corticosteroid induced effects between neonatal groups 1 and 2. However, neither the subgroup analyses stratified by antenatal corticosteroid exposure nor the multiple linear regression analyses provided evidence that differences in antenatal corticosteroid exposure explained the reduced inflammasome activity observed in preterm infants. Although the influence of antenatal corticosteroids on inflammasome activity remains uncertain, the observed reductions in inflammasome/caspase-1 pathway functions likely reflect the physiological state of preterm infants in routine neonatal intensive care settings, where prenatal corticosteroid exposure is standard practice.

### 4.3 Relevance for early-onset sepsis

The reduced activation potential of inflammasome pathways may protect the host from excessive inflammation during the period of bacterial colonization after birth (42). However, this reduced innate immune activity may also increase the risk of systemic bacterial infection. Typical pathogens in early-onset sepsis, such as Gram-negative bacteria and Group B Streptococcus, are sensed by the innate immune system via the canonical NLRP3 inflammasome pathway (43). Additionally, the alternative NLRP3 activation occurs in human monocytes in response to TLR activation and proceeds without requiring an activation signal (19). Multiple TLRs can trigger the alternative NLRP3 activation suggesting this pathway broadly responds to the diverse pathogens encountered during bacteremia, thereby establishing a rapid, host-wide immune response (44). A range of pathogens, including *Staphylococcus aureus, Listeria monocytogenes*, *Pseudomonas aeruginosa*, and *Burkholderia* spp., are additionally recognized via AIM2- and/or NLRC4-inflammasome pathways (45, 46). Beside the overall reduced activity of canonical NLRP3, AIM2, and NLRC4 pathways, reduced activity of the alternative NLRP3 inflammasome pathway is particularly important in weakening the early immune response during bacteremia, potentially contributing to the significantly higher incidence and mortality of culture-proven early-onset sepsis in preterm infants compared to term infants (47, 48).

### 4.4 Implications for inflammasome-mediated diseases

The reduced activation of inflammasome/caspase-1 pathways in preterm infants at birth observed in this study and prior reports (8, 9, 25) contrasts to the well-documented role of inflammasome activation in neonatal diseases such as retinopathy of prematurity, necrotizing enterocolitis or BPD (49). This apparent paradox raises a critical question: What drives the transition from significantly reduced inflammasome/caspase-1 activity in preterm infants at birth to dysregulated IL-1β production in the early neonatal period?.

Although reduced activation of inflammasome/caspase-1 pathways is typically reported in preterm infants, some studies reported enhanced pathway activation: The ELGAN study observed elevated spontaneous expression of IL-1β in whole blood of preterm infants born at 23–24 weeks of gestation compared to those born at higher gestational ages (50). Twisselman et al. reported augmented canonical NLRP3 inflammasome activation in monocyte-derived macrophages obtained from cord blood of preterm infants, contrasting with findings in term infants and adults (51). The reasons for these discrepancies are unclear. Notably, the latter study employed a double-hit stimulation protocol involving hyperoxia and LPS, combined with prolonged incubation times of up to 24 h with LPS. The biological significance of this time interval on NLRP3 signaling remains to be determined and warrants investigation in future studies.

An alternative explanation is the rapid postnatal maturation of the innate immune system of preterm infants. Olin et al. demonstrated that despite distinct immune profiles at birth, pre- and full-term infants rapidly converge onto a shared developmental trajectory (52). Similarly, Sharma et al. observed an increase in IL-1β secretion in peripheral blood of preterm infants aged 7–28 days, approaching adult levels, thereby providing preliminary evidence for rapid postnatal maturation of innate immune responses during early infancy (8).

Liao et al. reported significantly higher IL-1β levels in tracheal aspirates of ELGAN infants with adverse outcome (death or BPD) compared to controls, indicating activation of inflammasome/caspase-1 pathways in the early neonatal period (7). These findings suggest that tissue-resident immune cells—particularly pulmonary macrophages—may exhibit functional differences in inflammasome/caspase-1 pathways compared to circulating leukocytes in whole blood. Environmental triggers such as oxygen toxicity, infection or mechanical ventilation may accelerate the postnatal maturation of immune cells, potentially driving dysregulated inflammasome activation in preterm infants.

### 4.5 Regulation of inflammasome/caspase-1 pathways

Although our study was not designed to analyze the regulation of the inflammasome/caspase-1 pathways in detail, we additionally measured the expression levels of six inflammasome-related genes *NLRP3*, *AIM2*, *NLRC4, ASC, CASP1* and *IL1β*. To minimize experimental bias, gene expression analysis was normalized using an *ACTB/GAPDH*-based normalization factor and additionally *RPLP0* as a single reference gene, based on validation data supporting their stability (18). Given that gene expression regulation is a fundamental mechanism for controlling signaling pathways, the age-dependent upregulation of all analyzed genes may contribute to the increase of inflammasome/caspase-1 activity observed during postnatal development. Despite significantly higher expression of *NLRP3*, *AIM2*, *ASC*, *CASP1*, and *IL1β* in adults compared to full-term infants, functional inflammasome activity did not differ in whole blood assays, indicating the role of additional regulatory mechanisms.

Given the heterogeneity of leukocyte subtypes in peripheral blood, differences in their relative proportions may contribute to variable inflammasome/caspase-1 activation potential. Nonclassical monocytes (CD14^low^/CD16^pos^) were found to be the predominant monocyte subset in preterm infants (8). This monocyte subset may represent an immature phenotype, as it failed to express intracellular pro-IL-1β following LPS stimulation. However, despite high intracellular pro-IL-1β expression in intermediate (CD14^high^/CD16^pos^) and classical (CD14^high^/CD16^neg^) monocytes from preterm infants, IL-1β secretion was markedly reduced from these subsets as well (8, 53). Thus, differences in leukocyte-subset composition may explain why total leukocyte count was not correlated with IL-1β secretion in the present study.

### 4.6 Limitations of the study

The current study has several limitations: (1) Despite the frequent use of neonatal cord blood in immunological studies involving infants, its validity as a surrogate for neonatal peripheral blood remains a subject of ongoing controversy. Based on immune cell composition and mass cytometry analyses, Olin et al. reported strong differences between cord blood and peripheral blood of infants (52), but the predictive value of neonatal cord blood remains unclear for functional analysis of inflammasome pathways. Future research should address the question whether these differences are mainly caused by differences between cord blood and peripheral blood, or if they are induced by dynamic changes of the immune system during the first days of life (52, 54). (2) Single-cell functional analyses of inflammasome/caspase-1 pathways were not performed; thus, our findings do not provide detailed insights into the cellular regulation of these pathways in preterm infants. Instead, we focused on bulk IL-1β release from whole blood, as systemic inflammatory responses may contribute to organ injury in this vulnerable population. (3) IL-1β was used as the sole functional readout. Although IL-1β is an important cytokine associated with inflammasome activation, inflammasome signaling may also involve IL-18 maturation, caspase-1 activation, and pyroptotic cell death. Therefore, our findings cannot determine whether the observed changes reflect activation of the broader inflammasome/caspase-1 pathway or a selective alteration in IL-1β production or release. Future studies should incorporate additional readouts to provide a more comprehensive assessment of inflammasome function. (4) Maternal or fetal complications leading to preterm delivery or cesarean section in term infants may have influenced the results of this study. However, owing to the limited sample size, we were unable to adequately control for these differences or perform reliable subgroup-adjusted analyses. An analysis of the indications for delivery across all study groups showed that they were represented across the subgroups, although their frequencies varied. (5) Unlike the term infants, nearly all preterm infants in this study had been exposed to antenatal corticosteroids. Although neither subgroup analyses stratified by antenatal corticosteroid exposure nor multiple linear regression analyses provided evidence that corticosteroid exposure accounted for the reduced inflammasome activity observed in preterm infants, the strong association between gestational age and corticosteroid exposure limits the extent to which their independent effects on IL-1β secretion can be distinguished in the present study.

## 5 Conclusion

Activity of the NLRP3, AIM2, and NLRC4 inflammasomes is significantly reduced in cord blood of preterm infants, particularly those with extremely low gestational age or birth weight, and correlates strongly with advancing gestational age, indicating a developmental trajectory of innate immune maturation. This functional hyporesponsiveness may protect against excessive inflammation in early life but may increase susceptibility to early-onset sepsis. The apparent paradox—reduced inflammasome activity at birth despite the high incidence of inflammasome-associated neonatal diseases such as bronchopulmonary dysplasia—suggests that dysregulated activation may emerge postnatally, potentially triggered by environmental stressors. Future research should investigate the dynamic evolution of inflammasome activity during the first days of life using single-cell resolution and longitudinal approaches to uncover mechanisms of immune maturation and inform targeted immunomodulatory strategies that balance infection defense with protection from inflammatory injury.

## Data Availability

The raw data supporting the conclusions of this article will be made available by the authors, without undue reservation.

## References

[1] Latz E Xiao TS Stutz A. Activation and regulation of the inflammasomes. Nat Rev Immunol. (2013) 13:397–411. 10.1038/nri3452 23702978 PMC3807999

[2] Yao J Sterling K Wang Z Zhang Y Song W. The role of inflammasomes in human diseases and their potential as therapeutic targets. Signal Transduct Target Ther. (2024) 9:10. 10.1038/s41392-023-01687-y 38177104 PMC10766654

[3] Winkler S Rösen-Wolff A. Rösen-Wolff A. Caspase-1: an integral regulator of innate immunity. Semin Immunopathol. (2015) 37:419–27. 10.1007/s00281-015-0494-4 26059719

[4] Omer M Melo AM Kelly L Dermott EJM Leahy TR Killeen O. Emerging role of the NLRP3 inflammasome and interleukin-1β in neonates. Neonatology. (2021) 117:545–54. 10.1159/000507584 33075792

[5] Northway WH Jr Rosan RC Porter DY. Pulmonary disease following respirator therapy of hyaline-membrane disease. N Engl J Med. (1967) 276:357–68. 10.1056/nejm196702162760701 5334613

[6] Bry K Hogmalm A Bäckström E. Mechanisms of inflammatory lung injury in the neonate: lessons from a transgenic mouse model of bronchopulmonary dysplasia. Semin Perinatol. (2010) 34:211–21. 10.1053/j.semperi.2010.02.006 20494738

[7] Liao J Kapadia VS Brown LS Cheong N Longoria C Mija D. The NLRP3 inflammasome is critically involved in the development of bronchopulmonary dysplasia. Nat Commun. (2015) 6:8977. 10.1038/ncomms9977 PMC6215764 26611836

[8] Sharma AA Jen R Kan B Sharma A Marchant E Tang A. Impaired NLRP3 inflammasome activity during fetal development regulates IL-1β production in human monocytes. Eur J Immunol. (2014) 45:238–49. 10.1002/eji.201444707 25311115 PMC4619041

[9] Glaser K Kern D Speer CP Schlegel N Schwab M Thome UH. Imbalanced inflammatory responses in preterm and term cord blood monocytes and expansion of the CD14 + CD16+ subset upon toll-like receptor stimulation. Int J Mol Sci. (2023) 24:4919. 10.3390/ijms24054919 36902350 PMC10002861

[10] Levy O Zarember KA Roy RM Cywes C Godowski PJ Wessels MR. Selective impairment of TLR-mediated innate immunity in human newborns: neonatal blood plasma reduces monocyte TNF- induction by bacterial lipopeptides, lipopolysaccharide, and imiquimod, but preserves the response to R-848. The Journal of Immunology. (2004) 173:4627–34. 10.4049/jimmunol.173.7.4627 15383597

[11] Levy O Coughlin M Cronstein BN Roy RM Desai A Wessels MR. The adenosine system selectively inhibits TLR-mediated TNF-α production in the human newborn. J Immunol. (2006) 177:1956–66. 10.4049/jimmunol.177.3.1956 16849509 PMC2881468

[12] Elahi S Ertelt JM Kinder JM Jiang TT Zhang X Xin L. Immunosuppressive CD71 + erythroid cells compromise neonatal host defence against infection. Nature. (2013) 504:158–62. 10.1038/nature12675 24196717 PMC3979598

[13] Mankan AK Dau T Jenne D Hornung V. The NLRP3/ASC/caspase-1 axis regulates IL-1β processing in neutrophils. Eur J Immunol. (2012) 42:710–5. 10.1002/eji.201141921 22213227

[14] Grinstein L Endter K Hedrich CM Reinke S Luksch H Schulze F. An optimized whole blood assay measuring expression and activity of NLRP3, NLRC4 and AIM2 inflammasomes. Clinical Immunology (Orlando, Fla). (2018) 191:100–9. 10.1016/j.clim.2017.11.011 29183866

[15] Brämer GR. International statistical classification of diseases and related health problems. Tenth Revision. World Heal Stat Q Rapp Trimest Stat Sanit Mond. (1988) 41:32–6. PMID: 3376487 3376487

[16] Blencowe H Cousens S Oestergaard MZ Chou D Moller A-B Narwal R. National, regional, and worldwide estimates of preterm birth rates in the year 2010 with time trends since 1990 for selected countries: a systematic analysis and implications. Lancet. (2012) 379:2162–72. 10.1016/s0140-6736(12)60820-4 22682464

[17] Coll RC Robertson AAB Chae JJ Higgins SC Muñoz-Planillo R Inserra MC. A small-molecule inhibitor of the NLRP3 inflammasome for the treatment of inflammatory diseases.—pubMed—nCBI. Nat Med. (2015) 21:248–55. 10.1038/nm.3806 25686105 PMC4392179

[18] Hieronymus K Dorschner B Schulze F Vora NL Parker JS Winkler JL. Validation of reference genes for whole blood gene expression analysis in cord blood of preterm and full-term neonates and peripheral blood of healthy adults. Bmc Genomics. (2021) 22:489. 10.1186/s12864-021-07801-0 34193041 PMC8244134

[19] Gaidt MM Ebert TS Chauhan D Schmidt T Schmid-Burgk JL Rapino F. Human monocytes engage an alternative inflammasome pathway. Immunity. (2016) 44:833–46. 10.1016/j.immuni.2016.01.012 27037191

[20] Peters AMJ Bertram P Gahr M Speer CP. Reduced secretion of lnterleukin-1 and tumor necrosis factor-α by neonatal monocytes. Neonatology. (1993) 63:157–62. 10.1159/000243926 8324095

[21] Bessler H Komlos L Punsky I Ntambi JA Bergman M Straussberg R. CD14 Receptor expression and lipopolysaccharide-induced cytokine production in preterm and term neonates. Neonatology. (2001) 80:186–92. 10.1159/000047141 11585981

[22] Sharma AA Jen R Brant R Ladd M Huang Q Skoll A. Hierarchical maturation of innate immune defences in very preterm neonates. Neonatology. (2014) 106:1–9. 10.1159/000358550 24603545 PMC4556450

[23] Anderson J Thang CM Thanh LQ Dai VTT Phan VT Nhu BTH. Immune profiling of cord blood from preterm and term infants reveals distinct differences in pro-inflammatory responses. Front Immunol. (2021) 12:777927. 10.3389/fimmu.2021.777927 34790206 PMC8591285

[24] Jennen L Weerdt LD Kouriannidi E Hanning N Cornejo AST Willen L. Cytokine levels in mother-infant pairs at term and preterm delivery. Pediatr Infect Dis J. (2025) 44:S61–5. 10.1097/inf.0000000000004666 39951077

[25] Marchant EA Kan B Sharma AA Van Zanten A Kollmann TR Brant R. Attenuated innate immune defenses in very premature neonates during the neonatal period. Pediatr Res. (2015) 78:492–7. 10.1038/pr.2015.132 26186294 PMC5059157

[26] Belderbos ME Levy O Meyaard L Bont L. Plasma-mediated immune suppression: a neonatal perspective. Pediatr Allergy Immunol. (2013) 24:102–13. 10.1111/pai.12023 23173652

[27] Yoshida T Takasaki I Kanegane H Inomata S Ito Y Tamura K. Intrauterine growth restriction modifies gene expression profiling in cord blood. Pediatr Int. (2014) 56:559–65. 10.1111/ped.12324 24612065

[28] Saugstad OD Kwinta P Wollen EJ Bik-Multanowski M Madetko-Talowska A Jagła M. Impact of antenatal glucocorticosteroids on whole-genome expression in preterm babies. Acta Paediatr. (2013) 102:349–55. 10.1111/apa.12166 23347050

[29] Schmidt AF Kannan PS Bridges JP Filuta A Lipps D Kemp M. Dosing and formulation of antenatal corticosteroids for fetal lung maturation and gene expression in rhesus macaques. Sci Rep. (2019) 9:9039–10. 10.1038/s41598-019-45171-6 31227752 PMC6588577

[30] Schlinzig T Johansson S Gunnar A Ekström TJ Norman M. Epigenetic modulation at birth—altered DNA-methylation in white blood cells after caesarean section. Acta Pædiatrica. (2009) 98:1096–9. 10.1111/j.1651-2227.2009.01371.x 19638013

[31] Curley A Stanworth SJ Willoughby K Fustolo-Gunnink SF Venkatesh V Hudson C. Randomized trial of platelet-transfusion thresholds in neonates. N Engl J Med. (2019) 380:242–51. 10.1056/nejmoa1807320 30387697

[32] Kirpalani H Ratcliffe SJ Keszler M Davis PG Foglia EE Te Pas A. Effect of sustained inflations vs intermittent positive pressure ventilation on bronchopulmonary dysplasia or death among extremely preterm infants: the SAIL randomized clinical trial. JAMA. (2019) 321:1165–75. 10.1001/jama.2019.1660 30912836 PMC6439695

[33] Goldenberg RL Culhane JF Iams JD Romero R. Epidemiology and causes of preterm birth. Lancet. (2008) 371:75–84. 10.1016/s0140-6736(08)60074-4 18177778 PMC7134569

[34] Weichert A Weichert TM Bergmann RL Henrich W Kalache KD Richter R. Factors for preterm births in Germany—an analysis of representative German data (KiGGS). Geburtshilfe Frauenheilkd. (2015) 75:819–26. 10.1055/s-0035-1557817 26366001 PMC4554493

[35] Moisiadis VG Matthews SG. Glucocorticoids and fetal programming part 1: outcomes. Nat Rev Endocrinol. (2014) 10:391–402. 10.1038/nrendo.2014.73 24863382

[36] Finer NN Carlo WA Walsh MC Rich W Gantz MG Laptook AR. Early CPAP versus surfactant in extremely preterm infants. N Engl J Med. (2010) 362:1970–9. 10.1056/nejmoa0911783 20472939 PMC3071534

[37] Wu L Zhou C Wu J Chen S Tian Z Du Q. Corticosterone inhibits LPS-induced NLRP3 inflammasome priming in macrophages by suppressing Xanthine oxidase. Mediat Inflamm. (2020) 2020:6959741. 10.1155/2020/6959741 PMC7251469 32508525

[38] Zhao Q Wu CS Fang Y Qian Y Wang H Fan YC. Glucocorticoid regulates NLRP3 in acute-on-chronic hepatitis B liver failure. Int J Méd Sci. (2019) 16:461–9. 10.7150/ijms.30424 PMC6428984 30911280

[39] Busillo JM Azzam KM Cidlowski JA. Glucocorticoids sensitize the innate immune system through regulation of the NLRP3 inflammasome*. J Biol Chem. (2011) 286:38703–13. 10.1074/jbc.m111.275370 21940629 PMC3207479

[40] Koerber RM Oberbeck S Kotthoff P Daecke SN Brossart P Held SAE. Dexamethasone induced dectin-1 activation enhances NLRP3 inflammasome activation. Front Immunol. (2025) 16:1656288. 10.3389/fimmu.2025.1656288 41019046 PMC12465279

[41] Molino A Terlizzi M Colarusso C Rossi A Somma P Saglia A. AIM2/IL-1*α*/TGF-*β* Axis in PBMCs from exacerbated chronic obstructive pulmonary disease (COPD) patients is not related to COX-2-dependent inflammatory pathway. Front Physiol. (2019) 10:1235. 10.3389/fphys.2019.01235 31632288 PMC6780005

[42] Levy O. Innate immunity of the newborn: basic mechanisms and clinical correlates. Nat Rev Immunol. (2007) 7:379–90. 10.1038/nri2075 17457344

[43] Costa A Gupta R Signorino G Malara A Cardile F Biondo C. Activation of the NLRP3 inflammasome by group B streptococci. J Immunol. (2012) 188:1953–60. 10.4049/jimmunol.1102543 22250086 PMC3273589

[44] Unterberger S Mullen L Flint MS Sacre S. Multiple TLRs elicit alternative NLRP3 inflammasome activation in primary human monocytes independent of RIPK1 kinase activity. Front Immunol. (2023) 14:1092799. 10.3389/fimmu.2023.1092799 37954581 PMC10639122

[45] Man SM Karki R Kanneganti T. AIM2 Inflammasome in infection, cancer, and autoimmunity: role in DNA sensing, inflammation, and innate immunity. Eur J Immunol. (2016) 46:269–80. 10.1002/eji.201545839 26626159 PMC4758349

[46] Bauer R Rauch I. The NAIP/NLRC4 inflammasome in infection and pathology. Mol Asp Med. (2020) 76:100863. 10.1016/j.mam.2020.100863 32499055

[47] Eichberger J Resch E Resch B. Diagnosis of neonatal sepsis: the role of inflammatory markers. Front Pediatr. (2022) 10:840288. 10.3389/fped.2022.840288 35345614 PMC8957220

[48] Hornik CP Fort P Clark RH Watt K Benjamin DK Smith PB. Early and late onset sepsis in very-low-birth-weight infants from a large group of neonatal intensive care units. Early Hum Dev. (2012) 88:S69–74. 10.1016/s0378-3782(12)70019-1 22633519 PMC3513766

[49] Green EA Garrick SP Peterson B Berger PJ Galinsky R Hunt RW. The role of the interleukin-1 family in complications of prematurity. Int J Mol Sci. (2023) 24:2795. 10.3390/ijms24032795 36769133 PMC9918069

[50] Leviton A Fichorova R Yamamoto Y Allred EN Dammann O Hecht J. Inflammation-related proteins in the blood of extremely low gestational age newborns. The contribution of inflammation to the appearance of developmental regulation. Cytokine. (2011) 53:66–73. 10.1016/j.cyto.2010.09.003 20934883 PMC2987520

[51] Twisselmann N Pagel J Künstner A Weckmann M Hartz A Glaser K. Hyperoxia/hypoxia exposure primes a sustained pro-inflammatory profile of preterm infant macrophages upon LPS stimulation. Front Immunol. (2021) 12:762789. 10.3389/fimmu.2021.762789 34868007 PMC8637891

[52] Olin A Henckel E Chen Y Lakshmikanth T Pou C Mikes J. Stereotypic immune system development in newborn children. Cell. (2018) 174:1277–1292.e14. 10.1016/j.cell.2018.06.045 30142345 PMC6108833

[53] Windhorst AC Heydarian M Schwarz M Oak P Förster K Frankenberger M. Monocyte signature as a predictor of chronic lung disease in the preterm infant. Front Immunol. (2023) 14:1112608. 10.3389/fimmu.2023.1112608 37090732 PMC10113536

[54] Lee AH Shannon CP Amenyogbe N Bennike TB Diray-Arce J Idoko OT. Dynamic molecular changes during the first week of human life follow a robust developmental trajectory. Nat Commun. (2019) 10:1092. 10.1038/s41467-019-08794-x 30862783 PMC6414553

