# Supplementary material for: Reduced NLRP3, AIM2 and NLRC4 inflammasome activity in cord blood of extremely preterm infants
Source: Front Pediatr. 2026 Sep 8;14:1909501. doi: 10.3389/fped.2026.1909501 (PMC13593884; doi:10.3389/fped.2026.1909501)
Supplement: Supplementary file 1 [file Datasheet1.pdf]

Supplementary Material

Table S1: Indications for preterm delivery (group 1) or cesarean section of full-term infants (group 2)

**Measure**	**Preterm infants (group 1, n = 87)**	**Full-term infants (group 2, n = 72)**
Maternal indications		
Previous cesarean Section, n (%)	0 (0)	35 (49)
Uterus myomatosus, n (%)	0 (0)	2 (3)
HELLP, n (%)	13 (15)	0 (0)
Pre-eclampsia, n (%)	3 (4)	1 (1)
Placenta praevia, vaginal bleeding n (%)	1 (1)	6 (8)
Previous maternal abd./thor. Surgery, n (%)	0 (0)	2 (3)
Cephalopelvic disproportion, n (%)	0 (0)	4 (6)
Psychological Problems, n (%)	1 (1)	0 (0)
Fetal indications		
Breech/transverse position n (%)	0 (0)	19 (26)
Monochorionic and monoamniotic n (%)	5 (6)	0 (0)
Spontaneous (preterm) labor n (%)	19 (22)	0 (0)
Premature rupture of membranes n (%)	16 (18)	0 (0)
Pathological doppler ultrasound n (%)	20 (23)	2 (3)
Pathological CTG n (%)	6 (7)	1 (1)
Polyhydramnion of unknown cause n (%)	1 (1)	0 (0)
Umbilical cord prolaps n (%)	1 (1)	0 (0)
Prolapsed amniotic sac n (%)	1 (1)	0 (0)

Table S2: Sample sizes and group characteristics in the NLRP3 inflammasome analysis.

**Measure**	**Preterm infants (group 1, n = 34)**			**Full-term infants (group 2, n = 32)**	**p-value**
Infant sex<br>*Female, n (%)*<br>*Male, n (%)*	 20 (59)<br>14 (41)			 12 (37.5)<br>20 (62.5)	*0.09*
Birth weight <10. Perc., n (%)	10 (29)			5 (16)	*0.24*
	**Subgroups (gestational age)**				
	**<28+0**	**28+0 - 31+6**	**32+0 - 36+6**		
Infant sex<br>*Female, n (%)*<br>*Male, n (%)*	 2 (33)<br>4 (66)	 5 (42)<br>7 (58)	 13 (81)<br>3 (19)	 12 (37.5)<br>20 (62.5)	*0.027*
Maternal age, mean (range), years	30.3 (27-37)	31.0 (24-39)	33.2 (26-41)	34.5 (25-42)	*0.38*
Gestational age, mean (range) in weeks	26+2 (24+4-27+5)	30+0 (28+1-31+3)	33+6 (32+0-36+5)	38+3 (37+0-41+3)	*0.0001*
Birth weight, mean (range), g<br><br>*ELBW (<1000 g), n (%)*<br>*VLBW (1000-1500 g), n (%)*<br>*LBW (<2500 g), n (%)*<br>*≥2500 g, n (%)*	630 (380-1110)<br><br>5 (80)<br>1 (20)<br>0<br>0	1250 (680-1895)<br><br>2 (16,6)<br>8 (66,6)<br>2 (16,6)<br>0	1987 (1210-2640)<br><br>0 (0)<br>2 (12,5)<br>12 (75)<br>2 (12,5)	3288 (2300-4555)<br><br>0 (0)<br>0 (0)<br>2 (6)<br>30 (94)	*< 0.0001*
Birth weight <10. Perc., n (%)	4 (67)	2 (17)	4 (25)	5 (16)	*0.07*
Umbilical artery pH, mean (range)	7.29 (7.25-7.35)	7.30 (7.13-7.41)	7.32 (7.26-7.39)	7.33 (7.26-7.39)	*0.45*
Receipt of antenatal corticosteroids<br>*None, n (%)*<br>*Incomplete course, n (%)*<br>*Full course, n (%)*	 0 (0)<br>1 (20)<br>5 (80)	 1 (8,3)<br>1 (8,3)<br>10 (83,3)	 1 (6)<br>5 (31)<br>10 (63)	 32 (100)<br>0 (0)<br>0 (0)	*0.0001*
APGAR 10 min, mean, (range)	9.2 (9-10)	8.7 (7-10)	9.0 (6-10)	9.9 (9-10)	*0.001*

Table S3: Indications for preterm delivery (group 1) or cesarean section of full-term infants (group 2) included in the NLRP3 inflammasome analysis.

**Measure**	**Preterm infants (group 1, n = 34)**			**Full-term infants (group 2, n = 32)**
	**Subgroups (gestational age)**			
	**<28+0**	**28+0 - 31+6**	**32+0 - 36+6**	
Maternal indications				
Previous cesarean Section, n (%)	0 (0)	0 (0)	0 (0)	17 (53)
Uterus myomatosus, n (%)	0 (0)	0 (0)	0 (0)	0 (0)
HELLP, n (%)	2 (33)	1 (8)	1 (6)	0 (0)
Pre-eclampsia, n (%)	2 (33)	2 (17)	2 (13)	0 (0)
Placenta praevia, vaginal bleeding n (%)	0 (0)	1 (8)	0 (0)	3 (9)
Previous maternal abd./thor. Surgery, n (%)	0 (0)	0 (0)	0 (0)	1 (3)
Cephalopelvic disproportion, n (%)	0 (0)	0 (0)	0 (0)	1 (3)
Psychological Problems, n (%)	0 (0)	0 (0)	1 (6)	0 (0)
Fetal indications				
Breech/transverse position n (%)	0 (0)	0 (0)	2 (13)	10 (31)
Monochorionic and monoamniotic n (%)	0 (0)	0 (0)	2 (13)	0 (0)
Spontaneous (preterm) labor n (%)	1 (17)	0 (0)	2 (13)	0 (0)
Premature rupture of membranes n (%)	0 (0)	3 (25)	3 (19)	0 (0)
Pathological doppler ultrasound n (%)	1 (17)	4 (33)	3 (19)	0 (0)
Pathological CTG n (%)	0 (0)	1 (8)	0 (0)	0 (0)
Polyhydramnion of unknown cause n (%)	0 (0)	0 (0)	0 (0)	0 (0)
Umbilical cord prolaps n (%)	0 (0)	0 (0)	0 (0)	0 (0)
Prolapsed amniotic sac n (%)	0 (0)	0 (0)	0 (0)	0 (0)

Table S4: Sample sizes and group characteristics in the AIM2 inflammasome analysis.

**Measure**	**Preterm infants (group 1, n = 28)**			**Full-term infants (group 2, n = 21)**	**p-value**
Infant sex					*0.77*
Female, n (%)	14 (50)			9 (43)	
Male, n (%)	14 (50)			12 (57)	
Birth weight <10. Perc., n (%)	8 (29)			2 (10)	*0.30*
	**Subgroups (gestational age)**				
	**<28+0**	**28+0 - 31+6**	**32+0 - 36+6**		
Infant sex					*0.36*
Female, n (%)	2 (33)	3 (33)	9 (69)	9 (43)	
Male, n (%)	4 (67)	6 (67)	4 (31)	12 (57)	
Maternal age, mean (range), years	29.4 (19-42)	32.1 (23-39)	32.8 (26-43)	34.4 (26-41)	*0.65*
Gestational age, mean (range) in weeks	26+3 (24+6-27+5)	29+6 (28+1-31+6)	34+1 (32+0-36+6)	38+4 (37+0-41+3)	*<0.0001*
Birth weight, mean (range), g	739 (445-1110)	1066 (640-1445)	2105 (1505-3715)	3211 (2480-3800)	*<0.0001*
ELBW (<1000 g), n (%)	5 (83)	4 (44)	0 (0)	0 (0)	
VLBW (1000-1500 g), n (%)	1 (17)	5 (56)	0 (0)	0 (0)	
LBW (<2500 g), n (%)	0 (0)	0 (0)	11 (79)	1 (5)	
≥2500 g, n (%)	0 (0)	0 (0)	3 (21)	20 (95)	
Birth weight <10. Perc., n (%)	2 (33)	4 (44)	2 (15)	2 (10)	*0.12*
Umbilical artery pH, mean (range)	7.35 (7.25-7.47)	7.31 (7.13-7.47)	7.33 (7.22-7.38)	7.33 (7.27-7.37)	*0.73*
Receipt of antenatal corticosteroids					*0.05*
None, n (%)	0 (0)	0 (0)	3 (23)	21 (100)	
Incomplete course, n (%)	3 (50)	0 (0)	1 (8)	0 (0)	
Full course, n (%)	3 (50)	9 (100)	9 (69)	0 (0)	
APGAR 10 min, mean, (range)	8.6 (5-10)	8.9 (8-9)	8.9 (6-10)	9.9 (9-10)	*0.06*

Table S5: Indications for preterm delivery (group 1) or cesarean section of full-term infants (group 2) included in the AIM2 inflammasome analysis.

**Measure**	**Preterm infants (group 1, n = 28)**			**Full-term infants (group 2, n = 21)**
	**Subgroups (gestational age)**			
	**<28+0**	**28+0 - 31+6**	**32+0 - 36+6**	
Maternal indications				
Previous cesarean Section, n (%)	0 (0)	0 (0)	0 (0)	12 (57)
Uterus myomatosus, n (%)	0 (0)	0 (0)	0 (0)	0 (0)
HELLP, n (%)	3 (50)	0 (0)	1 (8)	0 (0)
Pre-eclampsia, n (%)	1 (17)	0 (0)	0 (0)	0 (0)
Placenta praevia, vaginal bleeding n (%)	0 (0)	0 (0)	0 (0)	2 (10)
Previous maternal abd./thor. Surgery, n (%)	0 (0)	0 (0)	0 (0)	0 (0)
Cephalopelvic disproportion, n (%)	0 (0)	0 (0)	0 (0)	1 (5)
Psychological Problems, n (%)	0 (0)	0 (0)	0 (0)	0 (0)
Fetal indications				
Breech/transverse position n (%)	0 (0)	0 (0)	0 (0)	4 (19)
Monochorionic and monoamniotic n (%)	0 (0)	3 (33)	2 (15)	0 (0)
Spontaneous (preterm) labor n (%)	1 (17)	1 (11)	2 (15)	0 (0)
Premature rupture of membranes n (%)	1 (17)	0 (0)	3 (23)	0 (0)
Pathological doppler ultrasound n (%)	0 (0)	4 (44)	4 (31)	2 (10)
Pathological CTG n (%)	0 (0)	0 (0)	1 (8)	0 (0)
Polyhydramnion of unknown cause n (%)	0 (0)	1 (11)	0 (0)	0 (0)
Umbilical cord prolaps n (%)	0 (0)	0 (0)	0 (0)	0 (0)
Prolapsed amniotic sac n (%)	0 (0)	0 (0)	0 (0)	0 (0)

Table S6: Sample sizes and group characteristics in the NLRC4 inflammasome analysis.

**Measure**	**Preterm infants (group 1, n = 29)**			**Full-term infants (group 2, n = 39)**	**p-value**
Infant sex<br>*Female, n (%)*<br>*Male, n (%)*	 16 (55)<br>13 (45)			 13 (33)<br>26 (67)	*0.07*
Birth weight <10. Perc., n (%)	9 (31)			7 (18)	*0.25*
	**Subgroups (gestational age)**				
	**<28+0**	**28+0 - 31+6**	**32+0 - 36+6**		
 Female, n (%)<br>Male, n (%)	 4 (80)<br>1 (20)	 4 (31)<br>9 (69)	 8 (73)<br>3 (27)	 13 (33)<br>26 (67)	*0.03*
Maternal age, mean (range), years	27.0 (19-34)	29.3 (23-38)	35.6 (26-43)	36.2 (29-41)	*0.002*
Gestational age, mean (range) in weeks	25+2 (24+0-27+1)	29+5 (28+1-31+6)	34+0 (32-36+6)	38+3 (37+0-41+3)	*<0.0001*
Birth weight, mean (range), g<br>*ELBW (<1000 g), n (%)*<br>*VLBW (1000-1500 g), n (%)*<br>*LBW (<2500 g), n (%)*<br>*≥2500 g, n (%)*	638 (560-825)<br>5 (100)<br>0 (0)<br>0 (0)<br>0 (0)	1083 (680-1450)<br>5 (38)<br>8 (62)<br>0 (0)<br>0 (0)	2228 (1800-3715)<br>0 (0)<br>0 (0)<br>8 (73)<br>3 (27)	3262 (1970-4430)<br>0 (0)<br>0 (0)<br>2 (5)<br>37 (95)	*<0.0001*
Birth weight <10. Perc., n (%)	2 (40)	5 (38)	2 (18)	7 (18)	*0.43*
Umbilical artery pH, mean (range)	7.32 (7.2-7.47)	7.32 (7.13-7.41)	7.33 (7.21-7.39)	7.31 (7.21-7.39)	*0.87*
Receipt of antenatal corticosteroids<br>*None, n (%)*<br>*Incomplete course, n (%)*<br>*Full course, n (%)*	 0 (0)<br>3 (60)<br>2 (40)	 0 (0)<br>1 (8)<br>12 (92)	 0 (0)<br>5 (45)<br>6 (55)	 39 (100)<br>0 (0)<br>0 (0)	*<0.0001*
APGAR 10 min, mean, (range)	8.2 (5-9)	8.8 (7-9)	9.1 (6-10)	9.8 (8-10)	*0.06*

Table S7: Indications for preterm delivery (group 1) or cesarean section of full-term infants (group 2) included in the NLRC4 inflammasome analysis.

**Measure**	**Preterm infants (group 1, n = 29)**			**Full-term infants (group 2, n = 39)**
	**Subgroups (gestational age)**			
	**<28+0**	**28+0 - 31+6**	**32+0 - 36+6**	
Maternal indications				
Previous cesarean Section, n (%)	0 (0)	0 (0)	0 (0)	19 (49)
Uterus myomatosus, n (%)	0 (0)	0 (0)	0 (0)	1 (3)
HELLP, n (%)	1 (20)	1 (8)	1 (9)	0 (0)
Pre-eclampsia, n (%)	1 (20)	2 (15)	0 (0)	1 (3)
Placenta praevia, vaginal bleeding n (%)	0 (0)	0 (0)	0 (0)	3 (8)
Previous maternal abd./thor. Surgery, n (%)	0 (0)	0 (0)	0 (0)	2 (5)
Cephalopelvic disproportion, n (%)	0 (0)	0 (0)	0 (0)	3 (8)
Psychological Problems, n (%)	0 (0)	0 (0)	1 (9)	0 (0)
Fetal indications				
Breech/transverse position n (%)	0 (0)	0 (0)	0 (0)	8 (21)
Monochorionic and monoamniotic n (%)	0 (0)	3 (23)	2 (18)	0 (0)
Spontaneous (preterm) labor n (%)	2 (40)	0 (0)	2 (18)	0 (0)
Premature rupture of membranes n (%)	0 (0)	1 (8)	2 (18)	0 (0)
Pathological doppler ultrasound n (%)	0 (0)	5 (38)	3 (27)	2 (5)
Pathological CTG n (%)	0 (0)	0 (0)	0 (0)	0 (0)
Polyhydramnion of unknown cause n (%)	0 (0)	1 (8)	0 (0)	0 (0)
Umbilical cord prolaps n (%)	0 (0)	0 (0)	0 (0)	0 (0)
Prolapsed amniotic sac n (%)	1 (20)	0 (0)	0 (0)	0 (0)

Table S8: Sample sizes and group characteristics in the gene expression analysis.

**Measure**	**Preterm infants (group 1, n = 40)**			**Full-term infants (group 2, n = 26)**	**p-value**
Infant sex					*0.08*
Female, n (%)	16 (40)			12 (46)	
Male, n (%)	24 (60)			14 (54)	
	**Subgroups (gestational age)**				
	**<28+0**	**28+0 - 31+6**	**32+0 - 36+6**		
Infant sex					*0.81*
Female, n (%)	3 (43)	6 (46)	7 (35)	12 (46)	
Male, n (%)	4 (57)	7 (54)	13 (65)	14 (54)	
Maternal age, mean (range), years	37.0 (27-47)	30.0 (27-33)	33.3 (32-36)	31.6 (28-49)	*0.02*
Gestational age, mean (range) in weeks	26+3 (23+2-27+3)	29+6 (28+1-31+6)	33+5 (32+0-36+6)	38+4 (37+0-41+3)	*<0.0001*
Birth weight, mean (range), g	603 (350-970)	1335 (825-1860)	2045 (1240-2630)	3060 (2310-4570)	*<0.0001*
				0 (0)	
ELBW (<1000g), n (%)	7 (100)	2 (15)	0 (0)	0 (0)	
VLBW (1000g-1500g), n (%)	0 (0)	9 (69)	1 (5)	5 (19)	
LBW (<2500g), n (%)	0 (0)	2 (15)	16 (80)	21 (81)	
≥2500g, n (%)	0 (0)	0 (0)	3 (15)		
Birth weight <10. Perc., n (%)	4 (57)	1 (8)	3 (15)	11 (42)	*0.02*
Umbilical artery pH, mean (range)	7.28 (7.20-7.40)	7.32 (7.13-7.43)	7.36 (7.31-7.40)	7.33 (7.31-7.36)	*0.43*
Receipt of antenatal corticosteroids					*< 0.0001*
None, n (%)	1 (14)	5 (38)	8 (40)	26 (100)	
Incomplete course, n (%)	0 (0)	1 (8)	0 (0)	0 (0)	
Full course, n (%)	6 (86)	7 (54)	12 (60)	0 (0)	
APGAR 10 min, mean, (range)	8.7 (7-10)	8.3 (8-9)	8.9 (8-10)	9.7 (9-10)	*0.0036*

Table S9: Indications for preterm delivery (group 1) or cesarean section of full-term infants (group 2) included in the gene expression analysis.

**Measure**	**Preterm infants (group 1, n = 40)**			**Full-term infants (group 2, n = 26)**
	**Subgroups (gestational age)**			
	**<28+0**	**28+0 - 31+6**	**32+0 - 36+6**	
Maternal indications				
Previous cesarean Section, n (%)	0 (0)	0 (0)	0 (0)	14 (54)
Uterus myomatosus, n (%)	0 (0)	0 (0)	0 (0)	2 (8)
HELLP, n (%)	3 (43)	0 (0)	3 (16)	0 (0)
Pre-eclampsia, n (%)	0 (0)	0 (0)	0 (0)	1 (4)
Placenta praevia, vaginal bleeding n (%)	0 (0)	0 (0)	0 (0)	0 (0)
Previous maternal abd./thor. Surgery, n (%)	0 (0)	0 (0)	0 (0)	1 (4)
Cephalopelvic disproportion, n (%)	0 (0)	0 (0)	0 (0)	0 (0)
Psychological Problems, n (%)	0 (0)	0 (0)	0 (0)	1 (4)
Fetal indications				
Breech/transverse position n (%)	0 (0)	0 (0)	0 (0)	5 (19)
Monochorionic and monoamniotic n (%)	0 (0)	0 (0)	0 (0)	0 (0)
Spontaneous (preterm) labor n (%)	2 (29)	7 (50)	4 (21)	0 (0)
Premature rupture of membranes n (%)	0 (0)	1 (7)	9 (47)	0 (0)
Pathological doppler ultrasound n (%)	1 (14)	5 (36)	2 (11)	2 (8)
Pathological CTG n (%)	0 (0)	1 (7)	1 (5)	0 (0)
Polyhydramnion of unknown cause n (%)	0 (0)	0 (0)	0 (0)	0 (0)
Umbilical cord prolaps n (%)	1 (14)	0 (0)	0 (0)	0 (0)
Prolapsed amniotic sac n (%)	0 (0)	0 (0)	0 (0)	0 (0)

Table S10: ***Pearson correlation coefficients among candidate predictors for each inflammasome pathway.*** *Birth weight was excluded from the regression models due to near-collinearity with gestational age (r = 0.918–0.943), and monocyte and neutrophil counts were excluded due to high correlation with total WBC (r = 0.82–0.96); total WBC was retained as a representative leukocyte parameter. Antenatal corticosteroid exposure was inversely correlated with gestational age but was deliberately retained as a predictor (see Methods). All coefficients shown are significant at $p < 0.01$.*

**Variable pair**	**Alt NLRP3**	**NLRP3**	**AIM2**	**NLRC4**
Gestational age ↔ birth weight	.941	.943	.933	.918
WBC ↔ monocytes	.935	.936	.892	.823
WBC ↔ neutrophils	.956	.956	.962	.955
Gestational age ↔ antenatal corticosteroids	−.779	−.785	−.720	−.813

Table S11: ***Multiple linear regression of ln(IL-1β) re-run with monocyte or neutrophil count substituted for total WBC.*** *First block: gestational age, corticosteroid dummies and the respective leukocyte parameter. β = standardized coefficient. In no model did the leukocyte parameter reach significance, and the gestational-age effect was unchanged, confirming that the choice of leukocyte predictor does not affect the conclusions.*

Pathway	Leukocyte term	n	R^2	Gestational age β (p)	Leukocyte β (p)
**Alt NLRP3**	WBC	49	.406	.751 (<.001)	.176 (.153)
	Monocytes	49	.419	.699 (.001)	.221 (.084)
	Neutrophils	49	.402	.771 (<.001)	.161 (.190)
**Canonical NLRP3**	WBC	50	.416	.591 (.005)	.069 (.567)
	Monocytes	50	.417	.573 (.007)	.081 (.520)
	Neutrophils	50	.412	.608 (.003)	.017 (.887)
**AIM2**	WBC	38	.758	1.159 (<.001)	.069 (.436)
	Monocytes	38	.770	1.147 (<.001)	.130 (.131)
	Neutrophils	38	.757	1.161 (<.001)	.059 (.505)
**NLRC4**	WBC	54	.417	.479 (.070)	.155 (.186)
	Monocytes	54	.405	.498 (.068)	.103 (.369)
	Neutrophils	54	.416	.473 (.073)	.155 (.195)

Table S12: ***Distribution of raw and natural-log-transformed IL-1β for each pathway.*** *Natural-log transformation reduced the right-skew of the raw data (raw skewness 1.29–2.53) and improved normality. KS, Kolmogorov–Smirnov.*

**Pathway**	**n**	**Raw skewness**	**Raw Shapiro–Wilk p**	**ln skewness**	**ln KS p**	**ln Shapiro–Wilk p**
Alternative NLRP3	61	2533	<.001	−0.472	.200	.217
Canonical NLRP3	62	1293	<.001	−0.839	.200	.006
AIM2	48	1626	<.001	−0.873	.200	.017
NLRC4	61	2022	<.001	−1.283	<.001	<.001

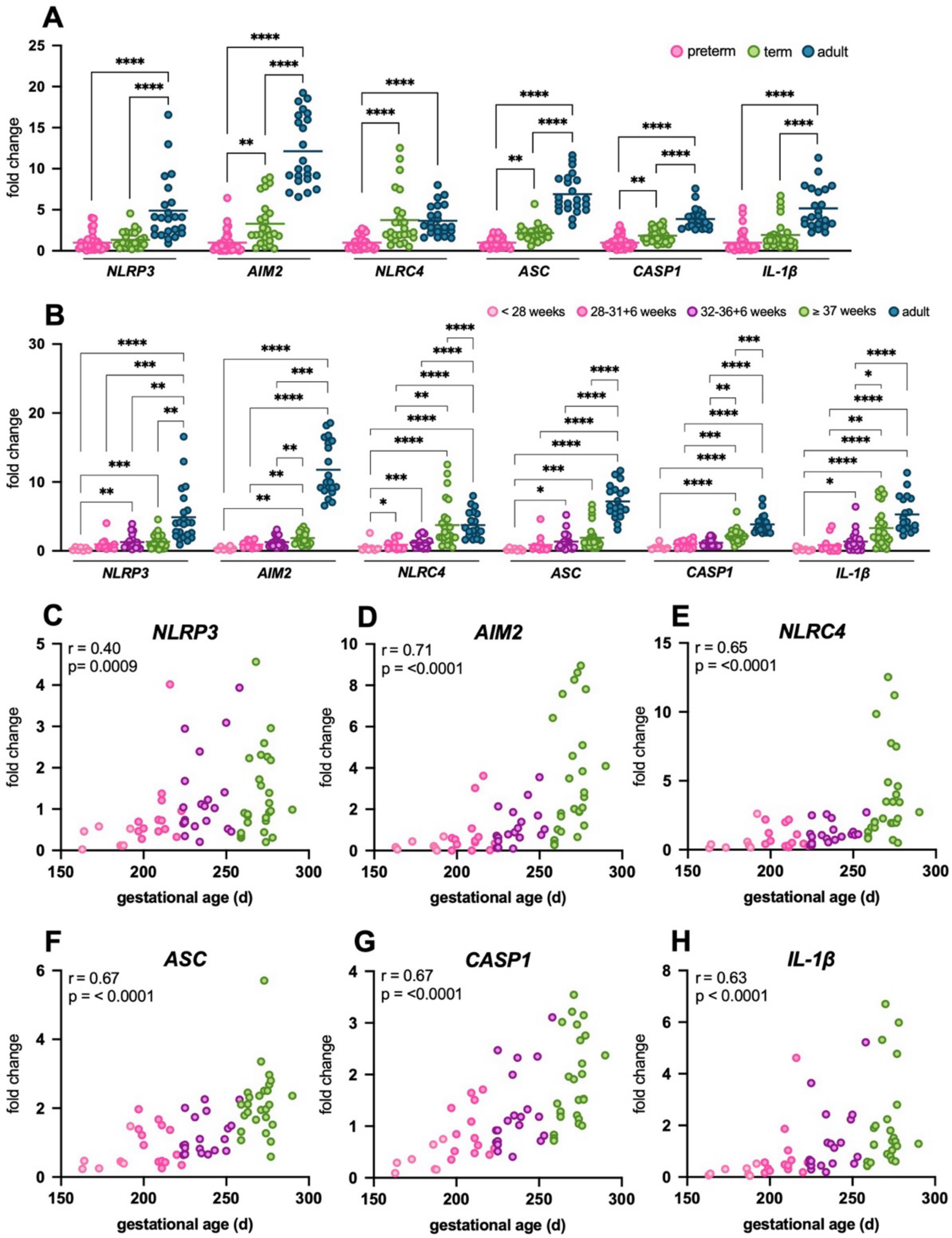

Figure S1: Expression of inflammasome related genes is reduced in infants. *(A-H) Non-stimulated, stabilized cord blood samples from pre- and full-term infants and peripheral blood of healthy adults were analyzed for gene expression of NLRP3, AIM2, NLRC4, ASC, CASP1, and IL1β using qPCR. Expression of target genes was normalized to the reference gene RPL0. (A) Data were analyzed for groups 1, 2, and 3. (B) Data were analyzed for all infants based on gestational age subgrouping, and the correlation between gene expression and gestational age was assessed (C-H). Detailed information on sample sizes and group characteristics is provided in supplementary tables S8 and S9.*

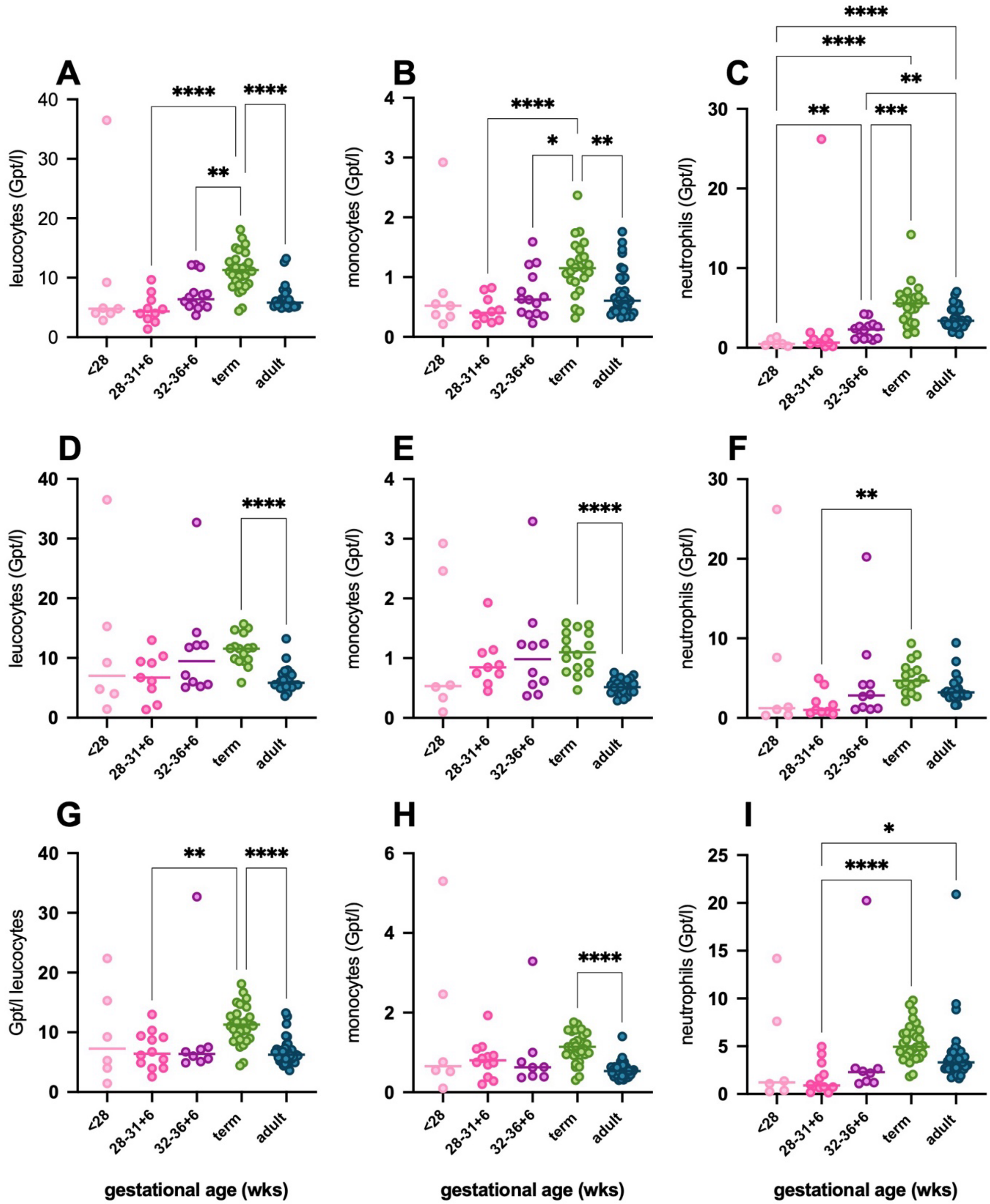

Figure S2. Cell counts in samples included in the different inflammasome analyses. *(A–C) Samples included in the NLRP3 inflammasome analysis. (D–F) Samples included in the AIM2 inflammasome analysis. (G–I) Samples included in the NLRC4 inflammasome analysis. Statistical analyses were performed using the Welch and Brown-Forsythe ANOVA test.*

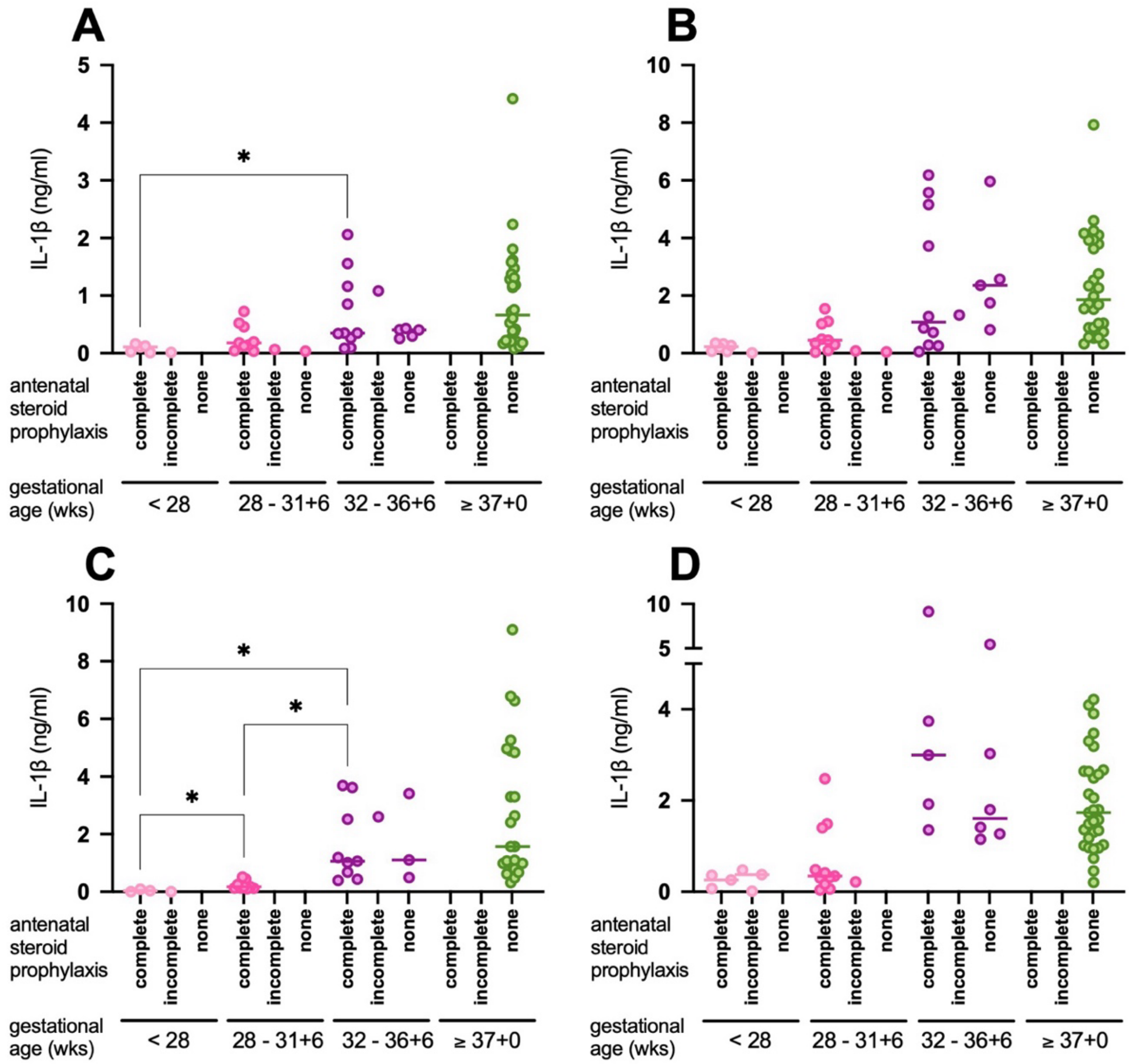

Figure S3: Analysis of antenatal steroid prophylaxis in the subgroups of different inflammasome analyses. *Cord blood of pre- and full-term infants and peripheral blood of healthy adult controls were stimulated (A) with LPS, (B) with LPS and ATP, (C) with LPS and transfection with poly(dA:dT) in the presence of the NLRP3 inhibitor MCC950, (D) with Salmonella typhimurium in the presence of the NLRP3 inhibitor MCC950. IL-1β concentrations in the supernatants were analyzed as surrogate marker of inflammasome/caspase-1 activation. Distribution of antenatal steroid (ANS) prophylaxis across the subgroups is shown additionally. (A, B) Sample sizes: <28+0: n=6, 28+0 – 31+6: n=12, ≥32+0: n=16). (C) Sample sizes: <28+0: n=6, 28+0 – 31+6: n=8, ≥32+0: n=13 (D) Sample sizes: <28+0: n=5, 28+0 – 31+6: n=13, ≥32+0: n=11. Subgroups of complete ANS prophylaxis were analyzed using the Welch and Brown-Forsythe ANOVA test.*

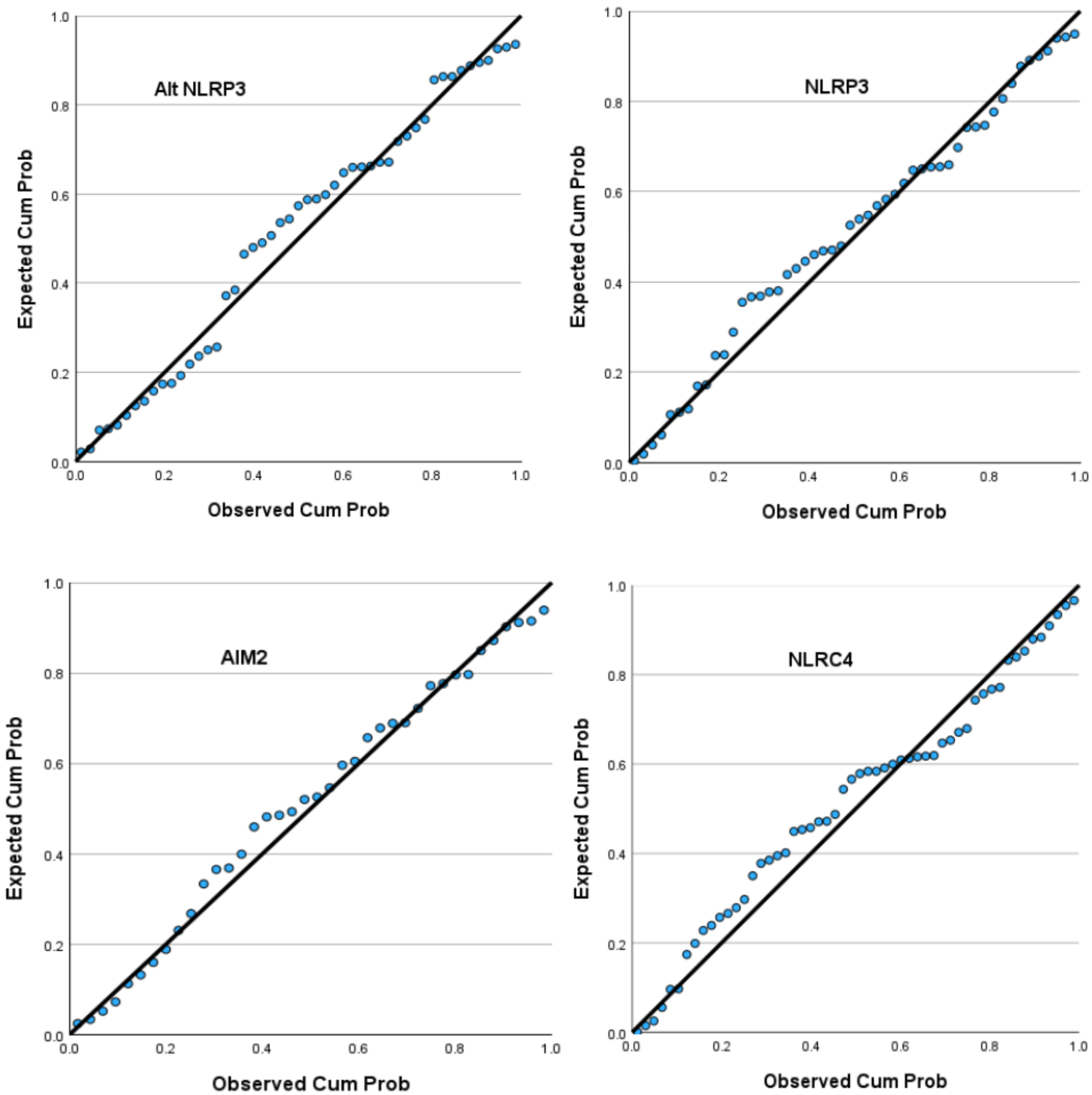

Figure S4: Regression diagnostics for the inflammasome models. *Normal P–P plots of the regression standardized residuals for the multiple linear regression models of ln(IL-1β) for the alternative NLRP3 (A), canonical NLRP3 (B), AIM2 (C) and NLRC4 (D) inflammasomes (combined infant cohort, primary WBC model+ umbilical artery pH). In all models the standardized residuals approximate the diagonal, supporting the assumption of residual normality underlying linear regression. Standardized residuals had a mean of 0 and a standard deviation of approx. 0.95, with largely symmetric ranges (alternative NLRP3 −2.03 to 1.52; canonical NLRP3 −2.67 to 1.64; AIM2 −1.98 to 1.54; NLRC4 −3.07 to 1.82). A single mild outlier (standardized residual −3.07) was present in the NLRC4 model.*
